# Supplementary material for: Demystifying “drop-outs” in single-cell UMI data
Source: Genome Biol. 2020 Aug 6;21:196. doi: 10.1186/s13059-020-02096-y (PMC7412673; doi:10.1186/s13059-020-02096-y)
Supplement: Supplementary file 1 — Additional file 1 Supplementary figures, tables, and text to support our claims [file 13059_2020_2096_MOESM1_ESM.pdf]

Supplementary Materials of "De-mystifying drop-outs in single cell UMI data"

This file includes figures and tables that supplement analyses in the main manuscript.

Section 1 introduces the data sets that are used for the analyses.

In Section 2, we study the relationship between zero proportions and gene means in the publicly available, labeled UMI data sets of Zheng2017, Azizi2016, Tabula Muris, Tung2017, Baron2016 (Figures SS1, S2, S3, S4, S5). We explore common cell types across different datasets to emphasize that the sampling noise affects different data sets and different cell types in the same way. We study the zero proportion and gene mean relationships in Figure S6 and S7 for data generated from CEL-seq2 and Drop-seq. In Drop-seq, the noise level was too high to assume the zero proportions follow the exponential curve relative to the gene mean. It is either that Drop-seq data sets have different noise structure from the 10X data sets, or in particular Macosko data of muscular retina cells have excessively high cellular heterogeneity. In Zhang2017 data, cells from different disease types show a layered pattern of zero inflation. This means that the Poisson noise model cannot separate the technical effect from the biological confounder of disease type.

In Section 3, we show why zero proportion is a better test statistic compared to gene variance and dispersion for cell-type heterogeneity using 4 data sets of Freytag2018, Zhengmix8eq, Tian2018, and Azizi2018.

Section 4 provides the details of the analysis that show immune-related genes are more zero-inflated than others. Table SS3 shows the number of genes present within each functional annotation for each data set. Table SS4 shows the result of enrichment analysis for AziziPatient09Rep01 data set to demonstrate that zero-inflated genes are particularly enriched in immune-related genes.

Section 5 compares the feature selection test statistic between HIPPO and scry package that uses deviance statistic. We in particular consider two scenarios with data sets with high UMI counts and low UMI counts, and show that in which case each method is more appropriate. Also, we compare the two feature selection methods in Figure S11 and S12 by looking at theri clustering performance, and for both high UMI count and low UMI count data, the results are similar. Figure S13 evaluates the use of MLE in the Poisson mixture model.

In Section 6, we show additional analyses that pre-processing steps before imputation and normalization lead to adversarial consequences in downstream analyses. Figure S14 shows that sequencing depths are confounded with the cell types, and normalization through size factors can either deflate or inflate the biological signals. Figure S15 expands the result of Figure 2 E by showing the differential expression analysis for known markers after DCA in two cases: on homogeneous cell population and on heterogeneous population. Figure S16 shows the similar result for both DCA and SAVER but transcriptome-wide statistics for log fold change, likelihood ratio, and p-values.

Section 7 evaluates the clustering performance for more data sets: Tian2018, Zhengmix4eq, Zhengmix4uneq, Zhengmix8eq, PBMC3k1, and PBMC4k1. Figure S17 shows the adjusted rand index for the available labeled data sets. Figure S18 shows the sequential visualization of HIPPO's clustering method for all of those data sets. Figure S19 evaluates the performance of generalized PCA (gPCA) that can account for the count structure directly [7].

Section 8 includes applications of HIPPO to two different data sets of cells from muscular brain tissue (1k Brain Cells from an E18 Mouse and 5k Cells from a combined cortex, hippocampus and subventricular zone of an E18 mouse). Figure S20 shows the clustering performance of HIPPO in two different data of un-labeled cells from brain tissue which are known to have a high level of heterogeneity. Figure S21 shows an example analysis pipeline implemented in HIPPO. Figure S22 compares two differential expression analysis results using Poisson likelihood and Gaussian approximation of the mean. Lastly, Figure S23 visualizes the hierarchical structure of the clustering result of HIPPO through an external tool "clustree". [13].

Lastly, Section 9 discusses in detail about the choice of zero-inflation statistic. It discusses the consequence of the simplification of the null distribution of the test statistic.

Contents

1 Table S1: Data Sets **4**

2 Zero Proportions in a homogeneous cell population follow a Poisson distribution. **5**
- 2.1 Figure S1: Azizi 2016 across different samples 5
- 2.2 Figure S2: Zheng2017 across different subsets 6
- 2.3 Figure S3: Tabula Muris across different subsets 7
- 2.4 Figure S4: Hi-Seq 8
- 2.5 Figure S5: in-Drop 9
- 2.6 Figure S6: CEL-Seq2 10
- 2.7 Figure S7: Drop-seq 11

3 Comparisons of zero proportions with gene variance as feature selection criteria. **12**
- 3.1 Figure S8: Gene variance for heterogeneous and homogeneous Cells 12
- 3.2 Table S2: Comparison of gene variance 12

4 Immune-related genes are more zero-inflated than other functional groups. **13**
- 4.1 Table S3: Blacklist genes 13
- 4.2 Table S4: Enrichment Analysis 14

5 Evaluation of Zero Inflation Test Statistic **15**
- 5.1 Figure S9: Case of Low Count Data 15
- 5.2 Figure S10: Case of High Count Data 16
- 5.3 Figure S11: Comparison of Two Feature Selection Methods on Clustering: Tian2018 17
- 5.4 Figure S12: Comparison of Two Feature Selection Methods on Clustering: Zhengmix4uneq 18
- 5.5 Figure S13: Comparison with MLE 19

6 Unwanted consequences of pre-processing when cell-type heterogeneity is not appropriately accounted for. **20**
- 6.1 Figure S14: Sequencing Depths 20
- 6.2 Figure S15: Imputation 21
- 6.3 Figure S16: Distribution of statistics 22

7 Comparisons of clustering performance using different tools **23**
- 7.1 Figure S17: ARI comparison with Seurat and Sctransform 23
- 7.2 Figure S18: Sequential visualization 24
- 7.3 Figure S19: Generalized PCA 25

8 Analysis with HIPPO **26**
- 8.1 Figure S20: HIPPO applied to Brain cells 26
- 8.2 Figure S21: HIPPO Analysis pipeline 28

8.3 Figure S22: Two Methods of Differential Expression 29
8.4 Figure S23: Tree structure of Hierarchical Clustering 29

9 Text S1: Details about z-score **30**

1 Table S1: Data Sets

ID	Data Set	Species	Protocol
10x	5KNeuron	Mouse Neuron	10x (v3.1) CR* 3.0.2
10x	10KHeart	Mouse Heart	10x (v3) CR 3.0.0
GSE111108 [10]	Tian2018	Human Cell Lines	10x Chromium
GSE115189[5]	Freytag2018	Human PBMC	10x (v2)
10x	1KNeuron	Mouse Neuron	10x (v2) CR 2.1.0
SRP073767[15]	Zhengmix4eq	Human PBMC	10x (v1) CR 1.1.0
SRP073767	Zhengmix4uneq	Human PBMC	10x (v1) CR 1.1.0
SRP073767	Zhengmix8eq	Human PBMC	10x (v1) CR 1.1.0
SRP073767	PBMC3k	Human PBMC	10x (v1) GemCode
SRP073767	PBMC4k	Human PBMC	10x (v1) Chromium
SRP073767	PBMC68k	Human PBMC	10x (v1) CR 1.1.0
GSE84133[2]	Baron2016	Human Pancreas	inDrop
GSE114724[1]	AziziPatient09Rep1	Human Breast Tumor	10x CR 2.1.1
GSE114724	AziziPatient09Rep2	Human Breast Tumor	10x CR 2.1.1
GSE114724	AziziPatient10Rep1	Human Breast Tumor	10x CR 2.1.1
GSE114724	AziziPatient11Rep1	Human Breast Tumor	10x CR 2.1.1
GSE114724	AziziPatient11Rep2	Human Breast Tumor	10x CR 2.1.1
SDY998[14]	Zhang2019	Human Joint Synovial	CEL-seq2
GSE63473[8]	Macosko2015	Mouse	Drop-seq
GSE77288 [12]	Tung2017	Human iPSC	HiSeq 2500
Tabula Muris [3]	Tabula Muris	Mouse	10x (v2)

Table S1: List of data sets used in the main text and supplementary materials. * CR: Cell Ranger

2 Zero Proportions in a homogeneous cell population follow a Poisson distribution.

2.1 Figure S1: Azizi 2016 across different samples

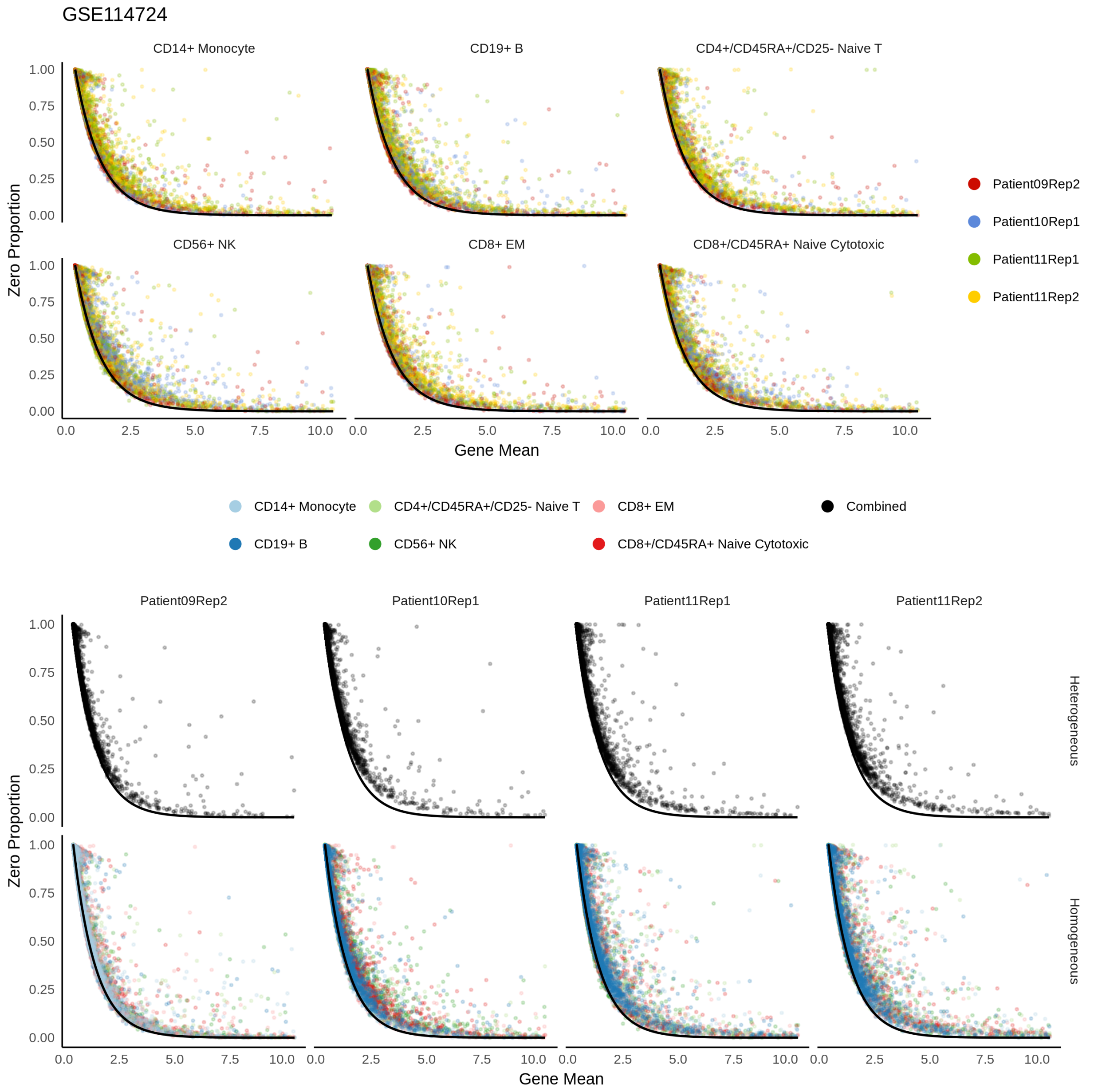

Figure S1: Zero proportions against gene means for Azizi data [1] for multiple samples and replicates. The top plot shows that the zero proportion matches the curve across the data sets for each cell type, while bottom plot across the cell types for each data set. The bottom plot also shows that the zero proportions are off the curve in heterogeneous cell populations. The consistent plots show that the sampling noise is the same across cell types and across data sets.

2.2 Figure S2: Zheng2017 across different subsets

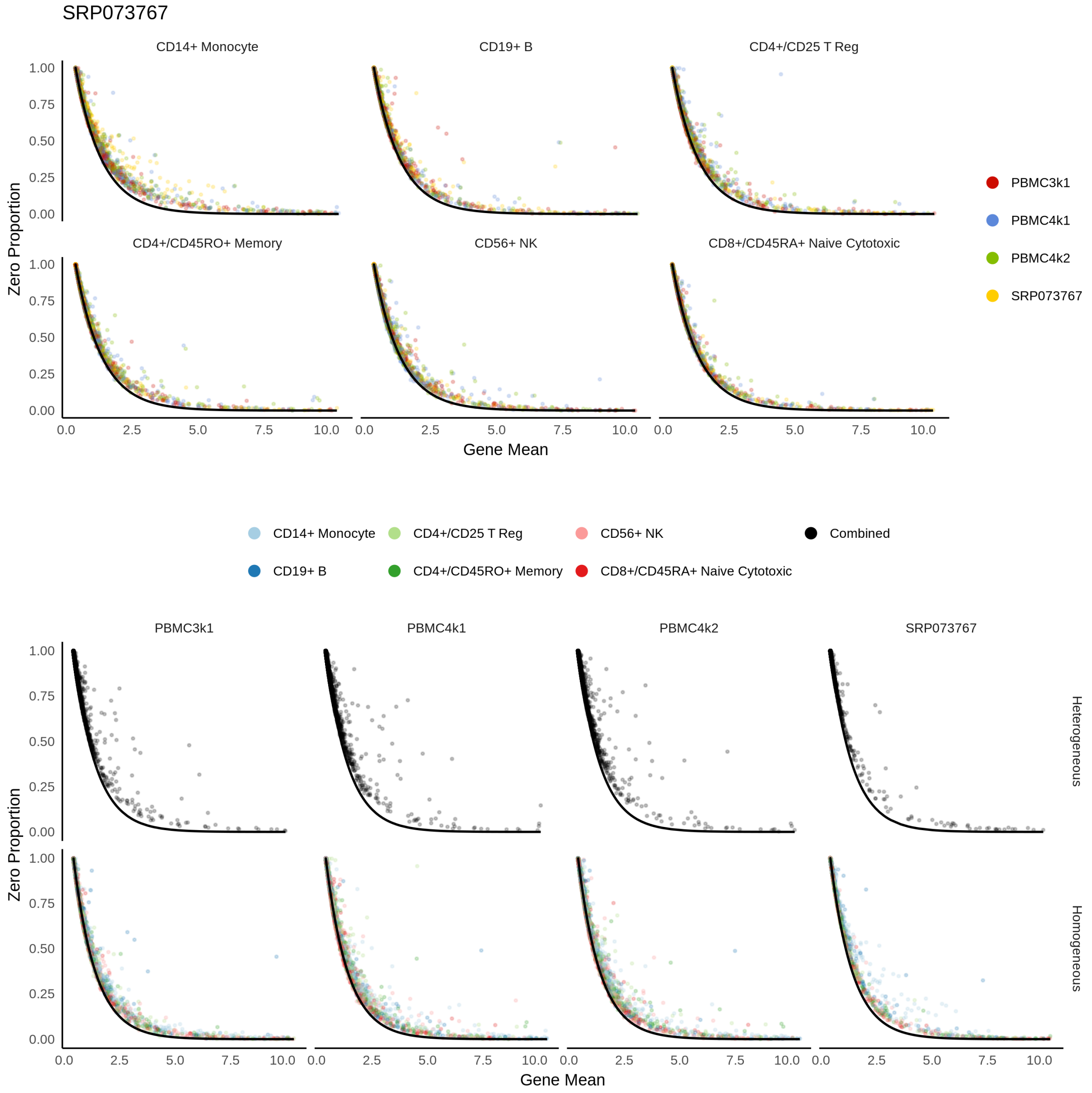

Figure S2: Same analysis as Figure S1 with Zheng2017 data [15]

2.3 Figure S3: Tabula Muris across different subsets

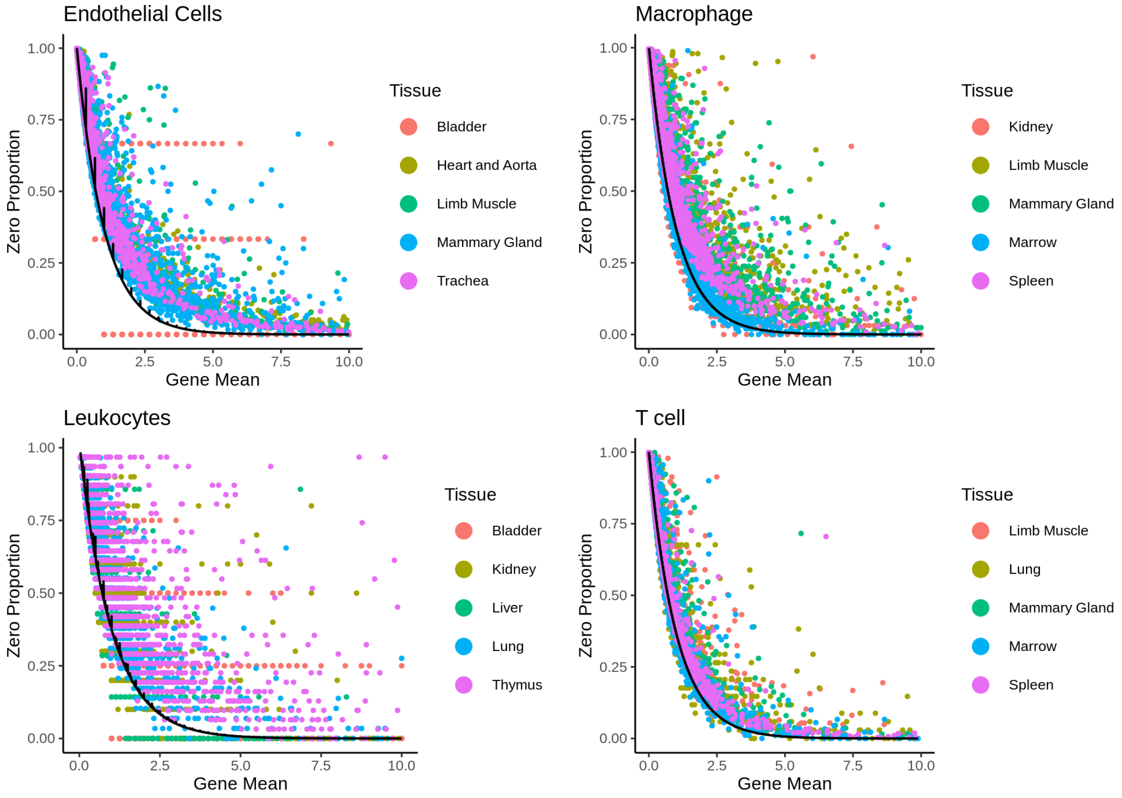

Figure S3: Same analysis as Figure S1 with Tabula Muris data [3] . The color codes are not tissue-specific to maximize visibility.

2.4 Figure S4: Hi-Seq

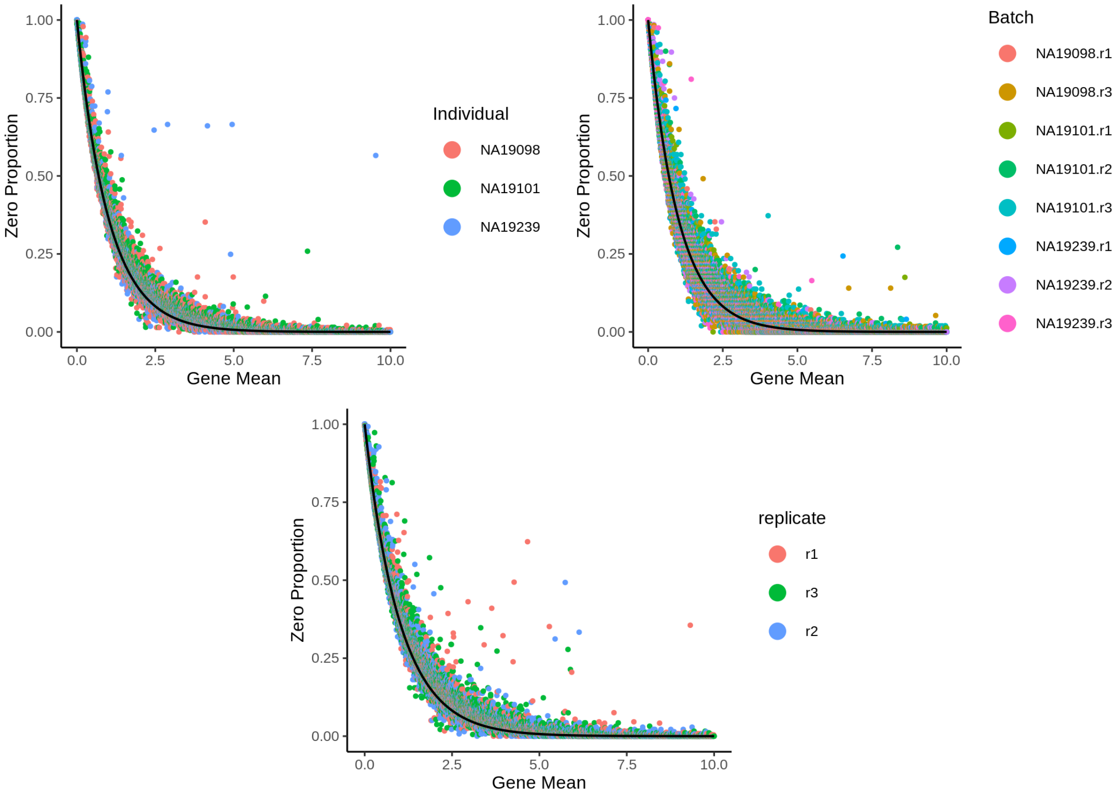

Figure S4: Results from Tung2017 [12] that uses Hi-Seq 2500. This data consists of homogeneous cell population of iPSC cell lines from three different individuals.

2.5 Figure S5: in-Drop

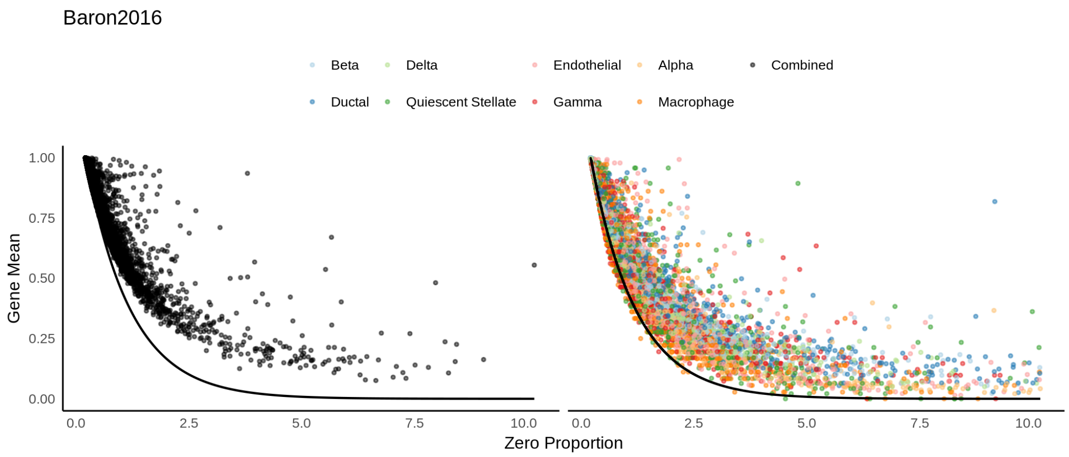

Figure S5: In-drop is promising that it can be modeled using Poisson.

2.6 Figure S6: CEL-Seq2

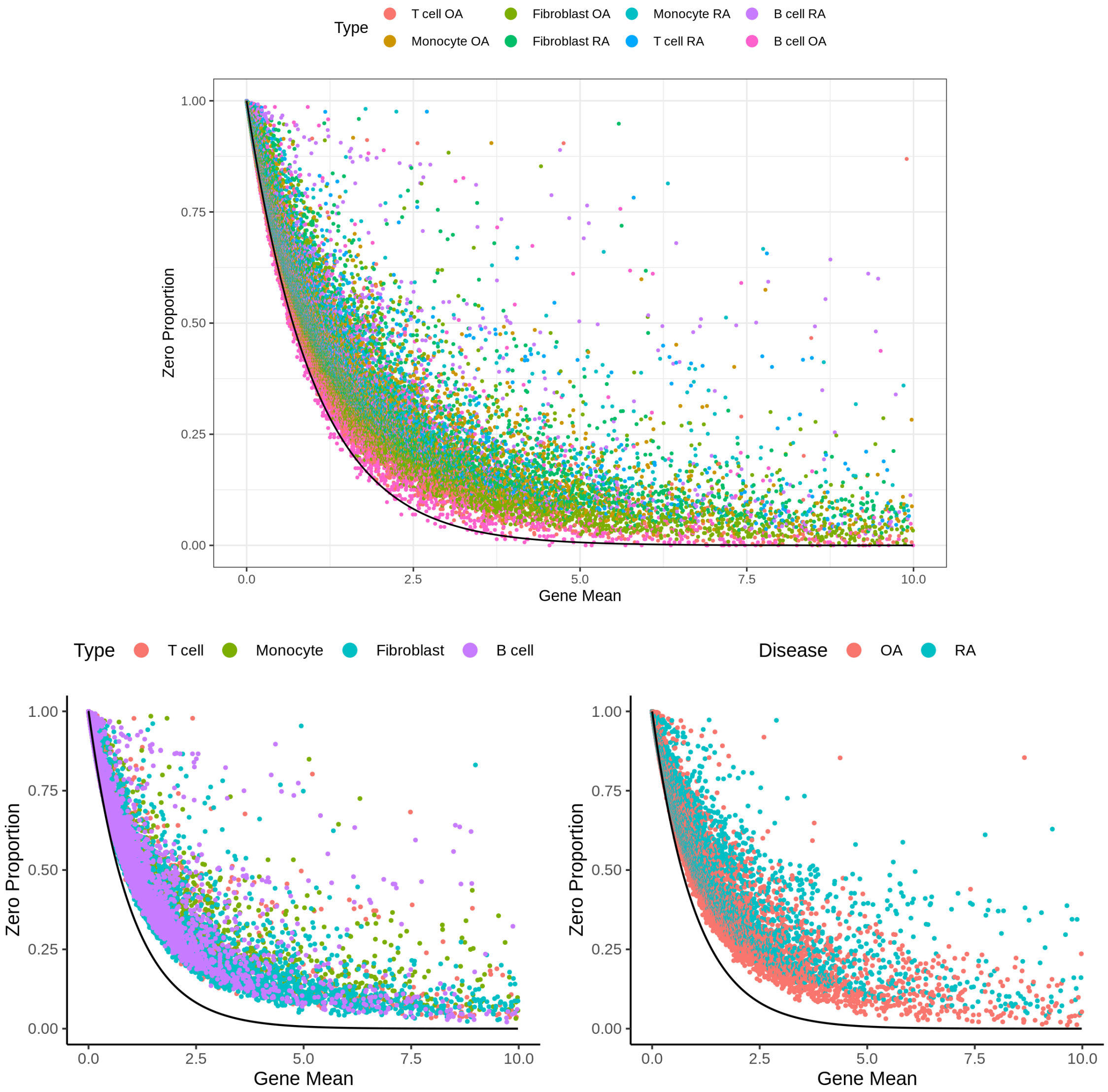

Figure S6: Results from Zhang2017 [14] that uses CEL-SEQ2 2500.

2.7 Figure S7: Drop-seq

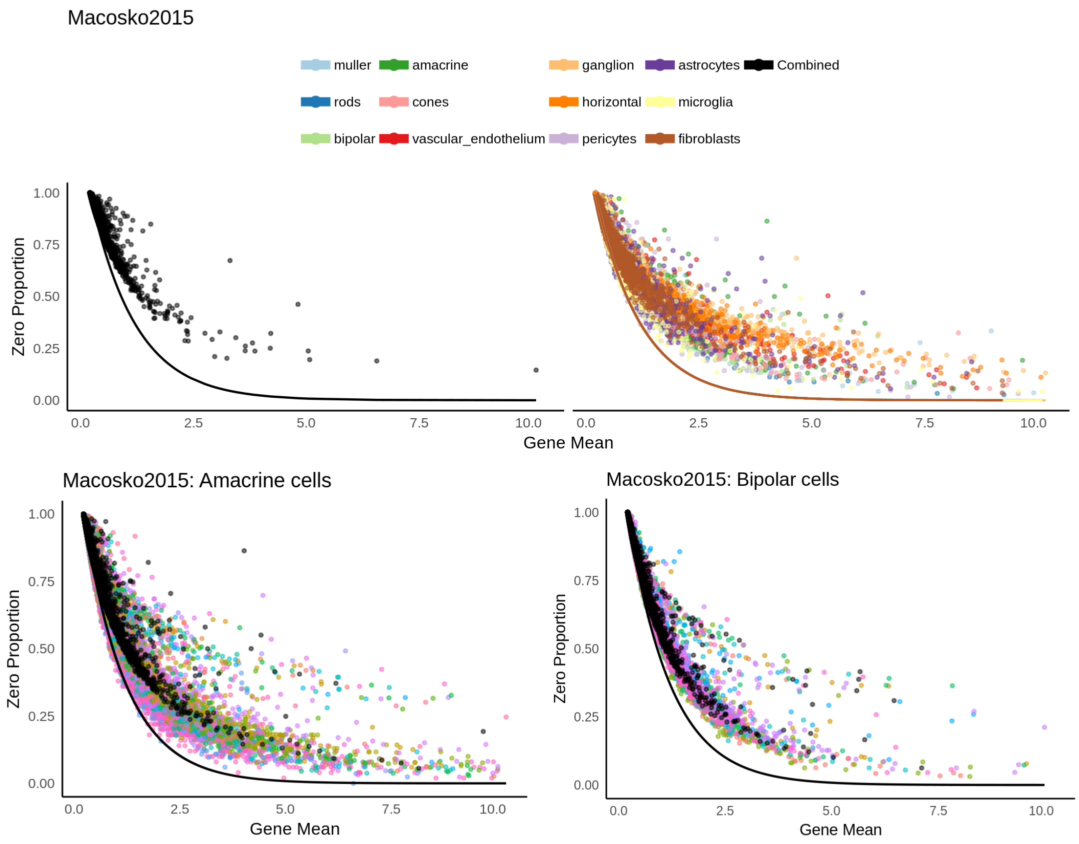

Figure S7: Results from Drop-seq[8]. However, the sampling noise of drop-seq data is too high, and zero-inflation element seems necessary. When amacrine cells were taken out and further clustered into subtypes, the noise level is closer to Poisson, so the culprit could be the particularly higher level of cell type diversity. The black points are plotted using heterogeneous cell population.

3 Comparisons of zero proportions with gene variance as feature selection criteria.

3.1 Figure S8: Gene variance for heterogeneous and homogeneous Cells

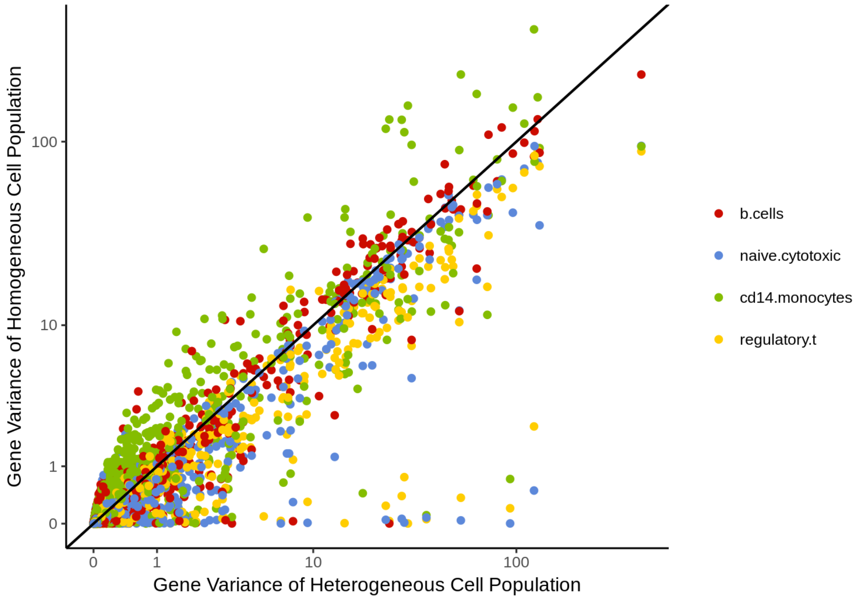

Figure S8: Gene variance for homogeneous cell population (y axis) and heterogeneous cell population (x-axis). For most genes, gene variance is similar for both heterogeneous and homogeneous cells. Further quantifications are provided in Table S2.

3.2 Table S2: Comparison of gene variance

Cell Type	Proportion of Genes with Higher Variance
B cells	0.4939857
Naive Cytotoxic	0.3700687
Monocytes	0.4969385
Regulatory T	0.4766347
Helper T	0.4620756
NK	0.7163943
Memory T	0.5348796
Naive T	0.3535812

Table S2: Proportion of genes that have higher variance in heterogeneous population than in homogeneous population. Using gene variance as feature selection would not be effective for detecting cellular heterogeneity.

4 Immune-related genes are more zero-inflated than other functional groups.

4.1 Table S3: Blacklist genes

	68K	Azizi09	Azizi10	Azizi11	Freytag	PBMC3k1	PBMC4k2
Antisense	2356	1701	1571	1537	269	305	329
HLA	23	21	21	21	29	31	21
IG-C	0	9	11	10	13	13	13
IG-C pseudo	0	1	2	2	5	4	6
IG-J gene	0	0	1	3	1	2	0
IG-V gene	0	60	49	77	61	77	67
IG-V pseudo	0	6	6	5	1	7	6
lincRNA	2202	1258	1140	1170	183	210	216
miRNA	0	1	2	1	1	2	0
misc RNA	1	0	0	0	96	190	0
Mt-rRNA	0	0	0	0	2	2	0
MT-tRNA	0	0	0	0	12	11	0
Polymorphic pseudo	0	6	6	6	11	11	7
Processed transcripts	3	58	52	55	86	88	46
Protein coding	15338	13684	13493	14080	12326	13025	13347
rRNA	0	0	0	0	14	18	0
Sense intronic	0	4	5	4	31	51	1
Sense overlapping	0	1	1	1	3	3	0
snoRNA	2	0	0	0	17	38	0
snRNA	0	0	0	0	71	134	0
TR-C gene	0	5	5	5	5	5	5
TR-J gene	0	1	2	46	0	0	0
TR-V gene	0	92	90	90	69	76	80
TR-V pseudogene	0	12	10	9	4	3	5

Table S3: Gene counts for each data set and each gene type for PBMC data [1, 5, 15]. Most of the genes are categorized as protein coding genes.

4.2 Table S4: Enrichment Analysis

Azizi Patient 9 Rep1		$z \leq 3$	$z > 3$	Proportion	χ^2_1 statistic
Before Clustering	Immune genes	98	109	52.66%	553.66
	Others	15476	1262	7.54%	$p < 2.2e-16$
After Clustering	Immune Genes	826	678	45.08%	10960
	Others	145941	5152	3.41%	$p < 2.2e-16$

Table S4: Azizi Patient9 Replication 1. Immune-related genes include HLA-gene, IG C gene, IG C pseudogene, IG V gene, IG V pseudogene, TR C gene, TR J gene, TR V gene, and TR V pseudogenes. The χ^2_1 statistic is computed through Pearson's chi squared test for independence of the two by two table. Clustering was performed using the true labels provided by the original paper [1]. Each gene is recorded once for each cell type, explaining the increase of the number of genes. By repeating the Pearson's chi squared test for the combined data for each cell type, we are implicitly assuming that each cell types are independent.

5 Evaluation of Zero Inflation Test Statistic

5.1 Figure S9: Case of Low Count Data

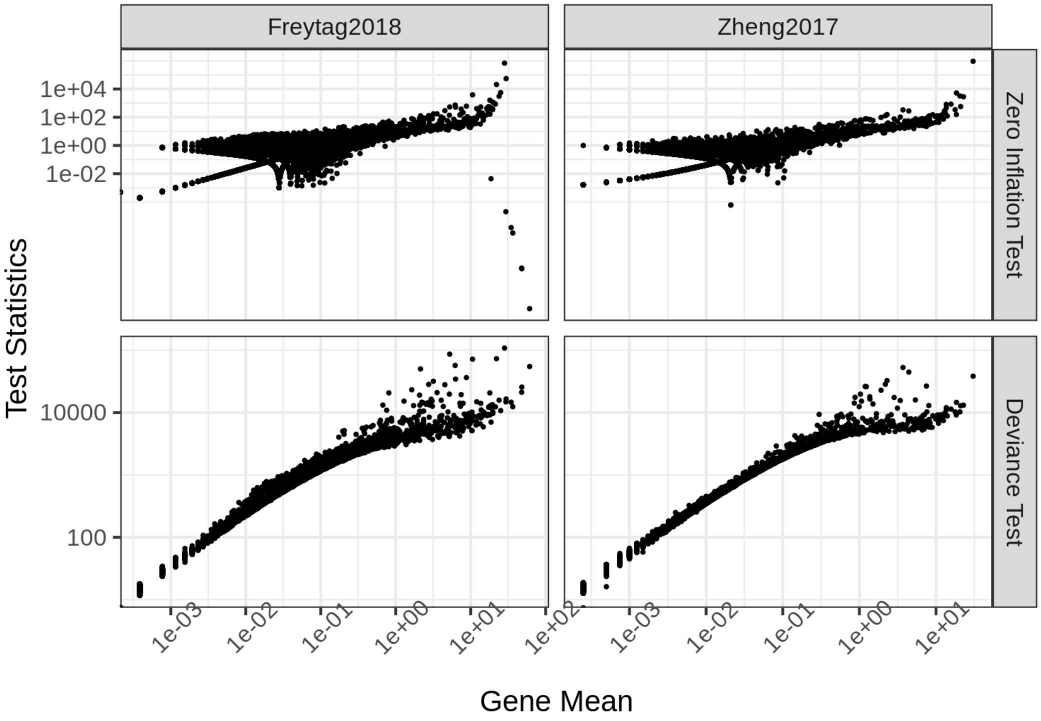

Figure S9: Comparison of the test statistics between the proposed package HIPPO and package scry [11]. The ordering of the statistic is similar between zero inflation test statistic and deviance statistic, although the zero inflation test does not take account into the entire distribution of the gene counts. There are a few genes that have high deviance but low zero inflation in Freytag data. Those cases occur when there are no zeros recorded across all the cells. Zero inflation test statistic becomes lower as gene mean increases.

5.2 Figure S10: Case of High Count Data

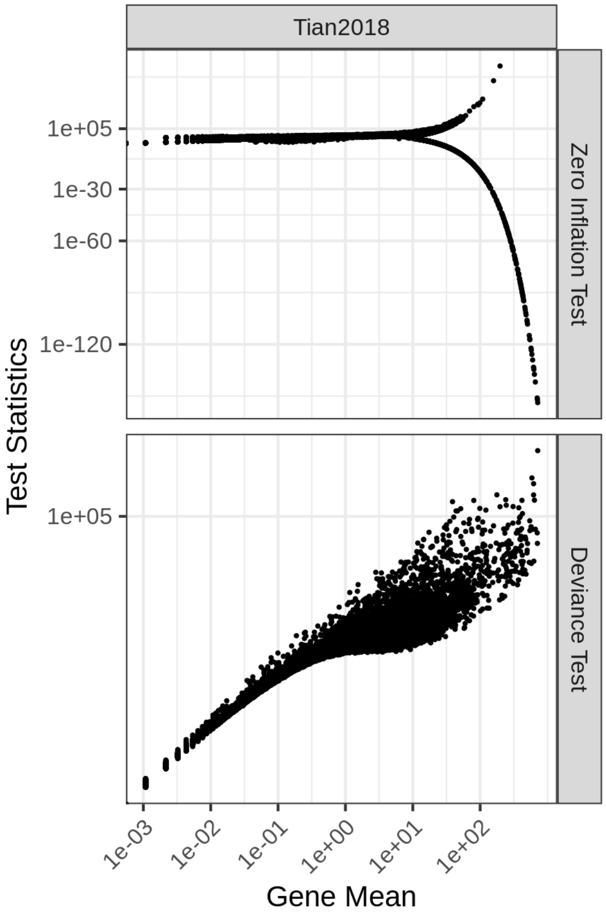

Figure S10: Same analysis as Figure S8 with Tian2018 data which has higher UMI counts. This analysis shows different relationship between zero inflation test and deviance test. When the mean counts become large, the zero inflation test statistic is either extremely low (there are no zeros recorded) or extremely high (there is at least 1 zero recorded). The problem is more severe when there are fewer cells as arguments for the test statistic are asymptotic. However, the HIPPO result shows that the zero-inflated genes still hold rich information for reliable clustering.

5.3 Figure S11: Comparison of Two Feature Selection Methods on Clustering: Tian2018

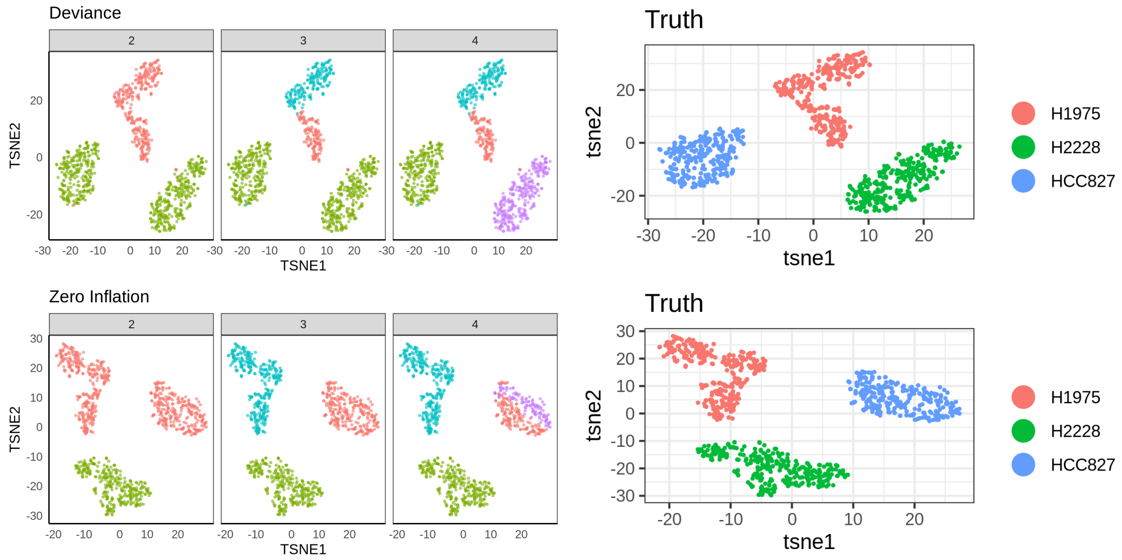

Figure S11: Clustering results for two feature selection methods - zero inflation and deviance with Tian2018 data that has high UMI counts. The truth labels are shown for both dimension reductions using different sets of features.

5.4 Figure S12: Comparison of Two Feature Selection Methods on Clustering: Zhengmix4uneq

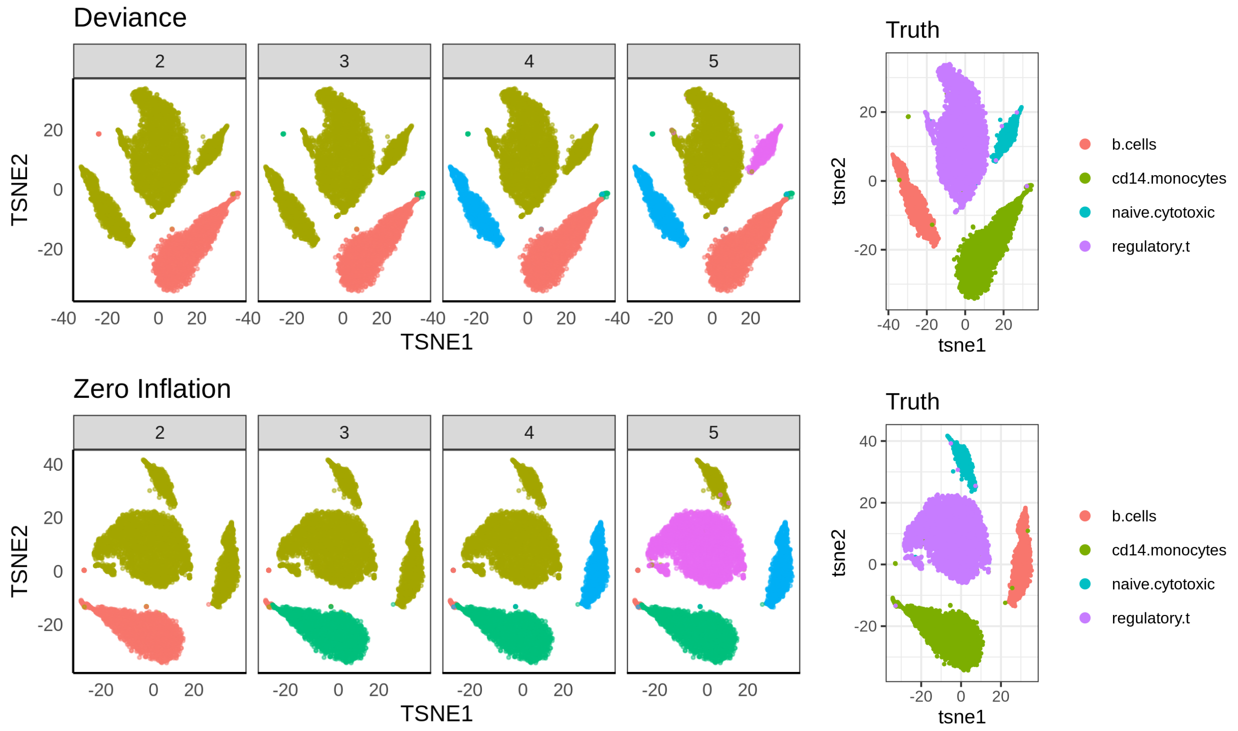

Figure S12: Clustering results for two feature selection methods in Zhengmix4uneq data that has low UMI counts. The truth labels are shown for both dimension reductions using different sets of features. The performance is very similar using two different methods.

5.5 Figure S13: Comparison with MLE

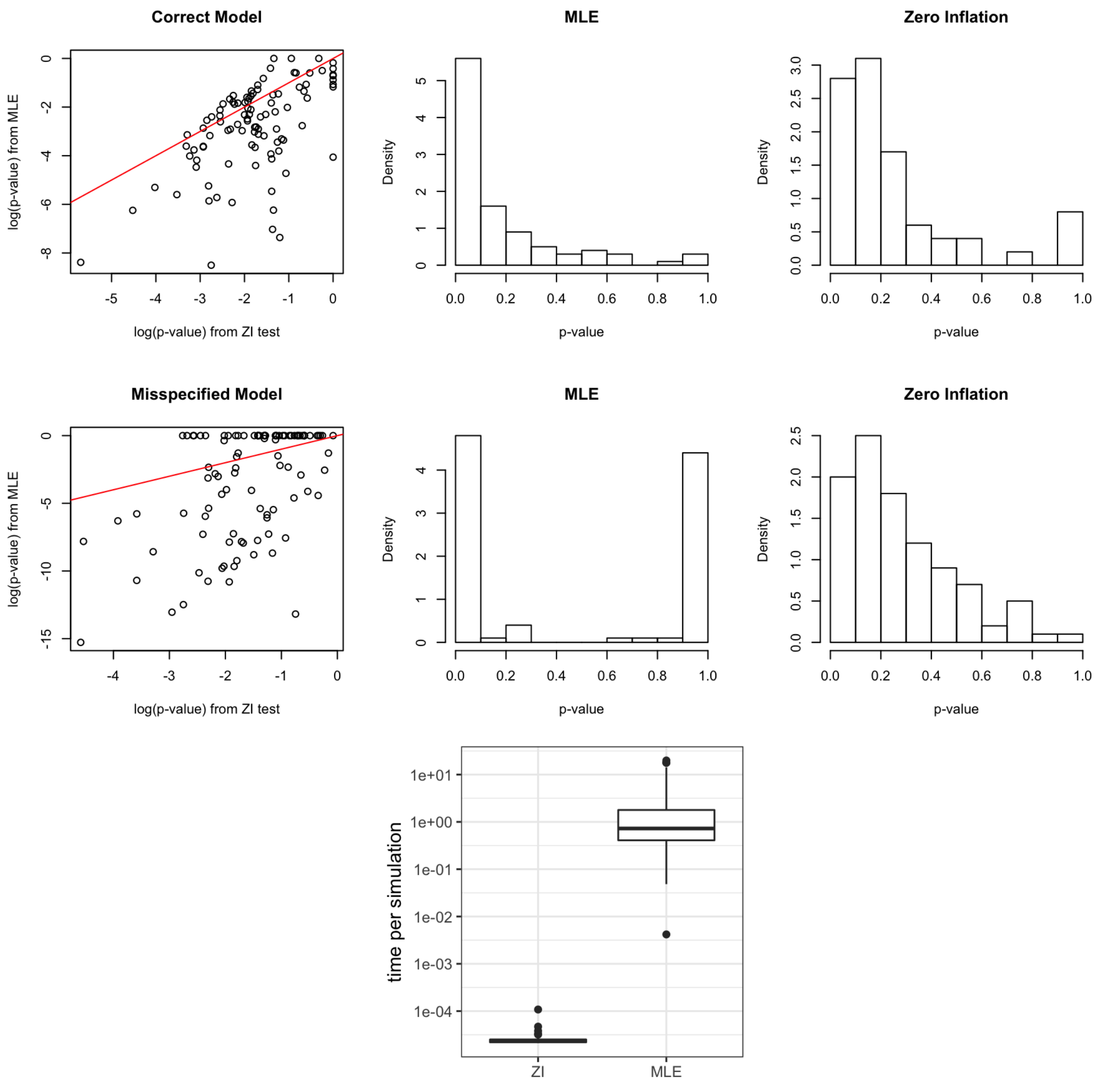

Figure S13: Comparison of using maximum likelihood estimates of Poisson mixture and using proposed zero inflation test. When data is generated from Negative Binomial, EM algorithm for mixture estimate often breaks down, leading to very unstable result. Moreover, EM algorithm is much more computationally intensive in the order of 10^4 to 10^5.

6 Unwanted consequences of pre-processing when cell-type heterogeneity is not appropriately accounted for.

6.1 Figure S14: Sequencing Depths

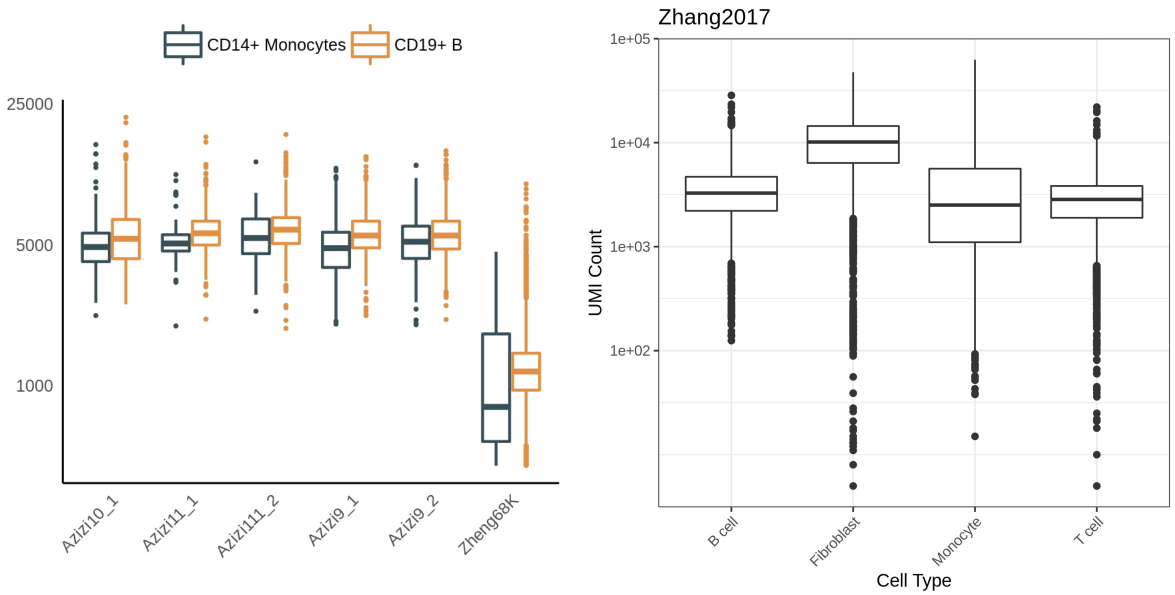

Figure S14: Sequencing depth of monocytes and B cells for 10X data sets on the left in PBMC and tumor data. Monocytes have consistently higher total UMI counts than B cells in these particular data sets, and forcing all the cells to have the same sequencing depth (size factor normalization) would either shrink the counts of B cells or inflate the counts of monocytes . On the right, same analysis is performed on Zhang2017 data, and cell types show slightly different patterns. This means that the UMI count difference among cell types do not necessarily expand to different tissues or organisms.

6.2 Figure S15: Imputation

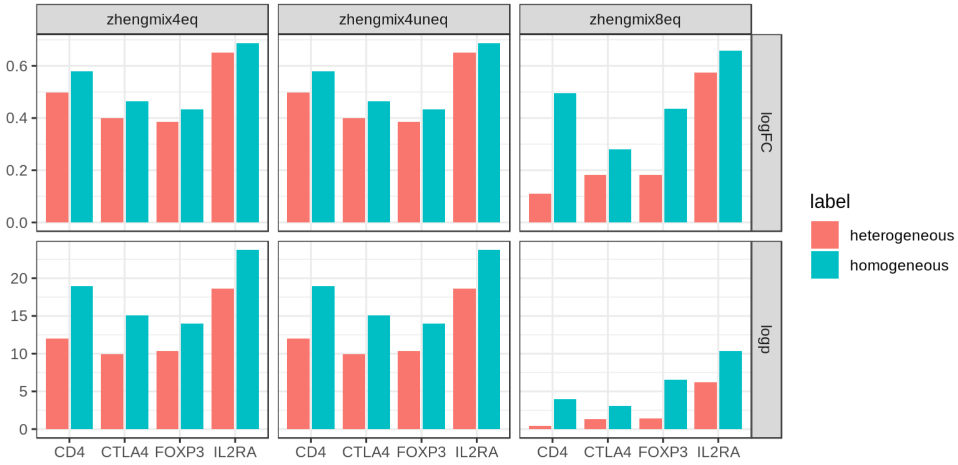

Figure S15: Extension of Figure 2 E in the main text. The log-fold change is consistently lower across data sets if DCA [4] is performed before the cell heterogeneity is accounted for.

6.3 Figure S16: Distribution of statistics

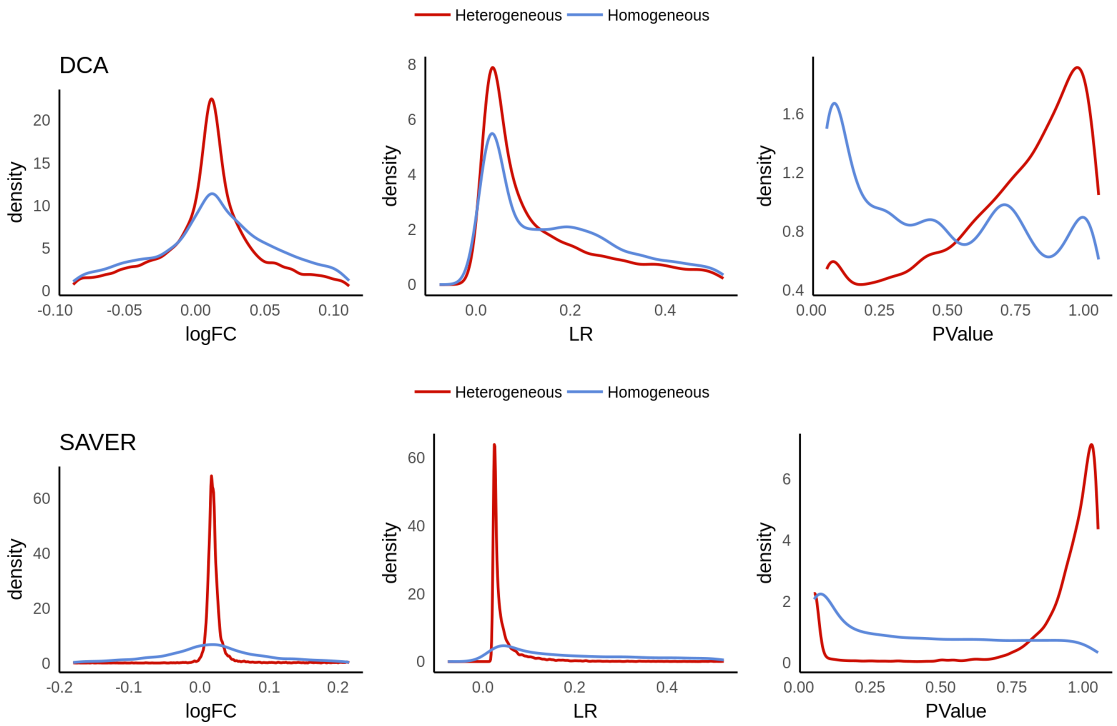

Figure S16: Extension of Figure S15. Overall distribution of various statistics (log fold change, likelihood ratio, and p-value) from differential expression test using edgeR's likelihood ratio test [9] after DCA [4] and SAVER [6]. Overall signal size is deflated if we perform imputation first.

7 Comparisons of clustering performance using different tools

7.1 Figure S17: ARI comparison with Seurat and Sctransform

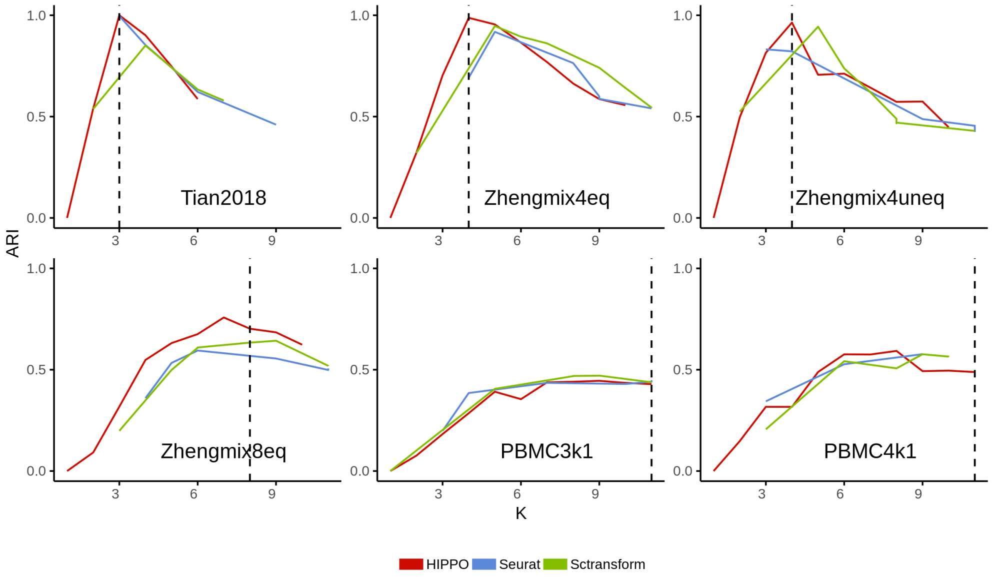

Figure S17: Adjusted Rand Index for various data sets comparing three methods. HIPPO tends to work at least as well as Sctransform and Seurat.

7.2 Figure S18: Sequential visualization

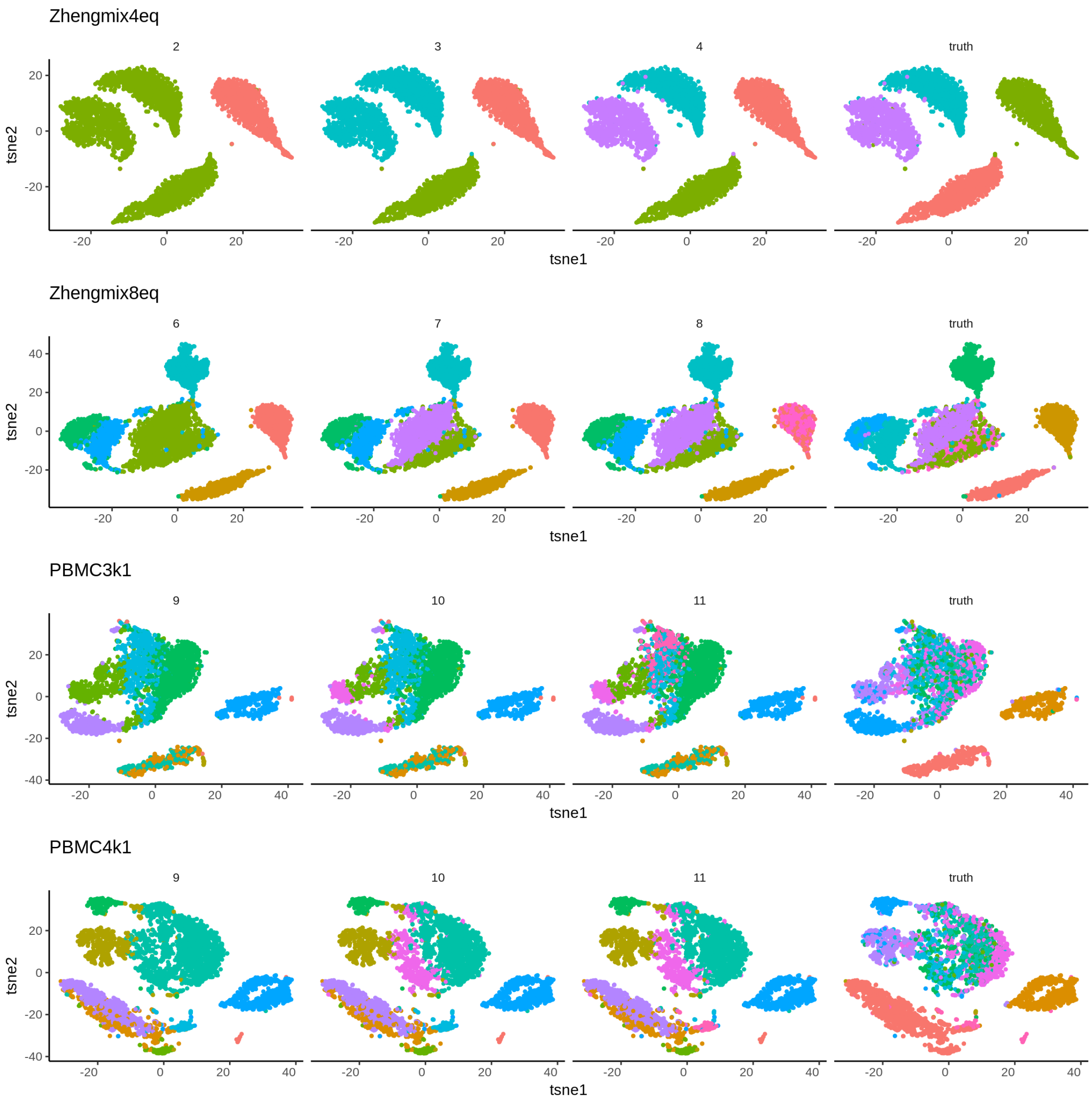

Figure S18: Visualization of the step-by-step clustering of HIPPO in various data sets. One drawback is that when it can no longer identify distinct clusters and forced to cluster into more groups, it can divide existing groups into subsets and drive down the adjusted rand index.

7.3 Figure S19: Generalized PCA

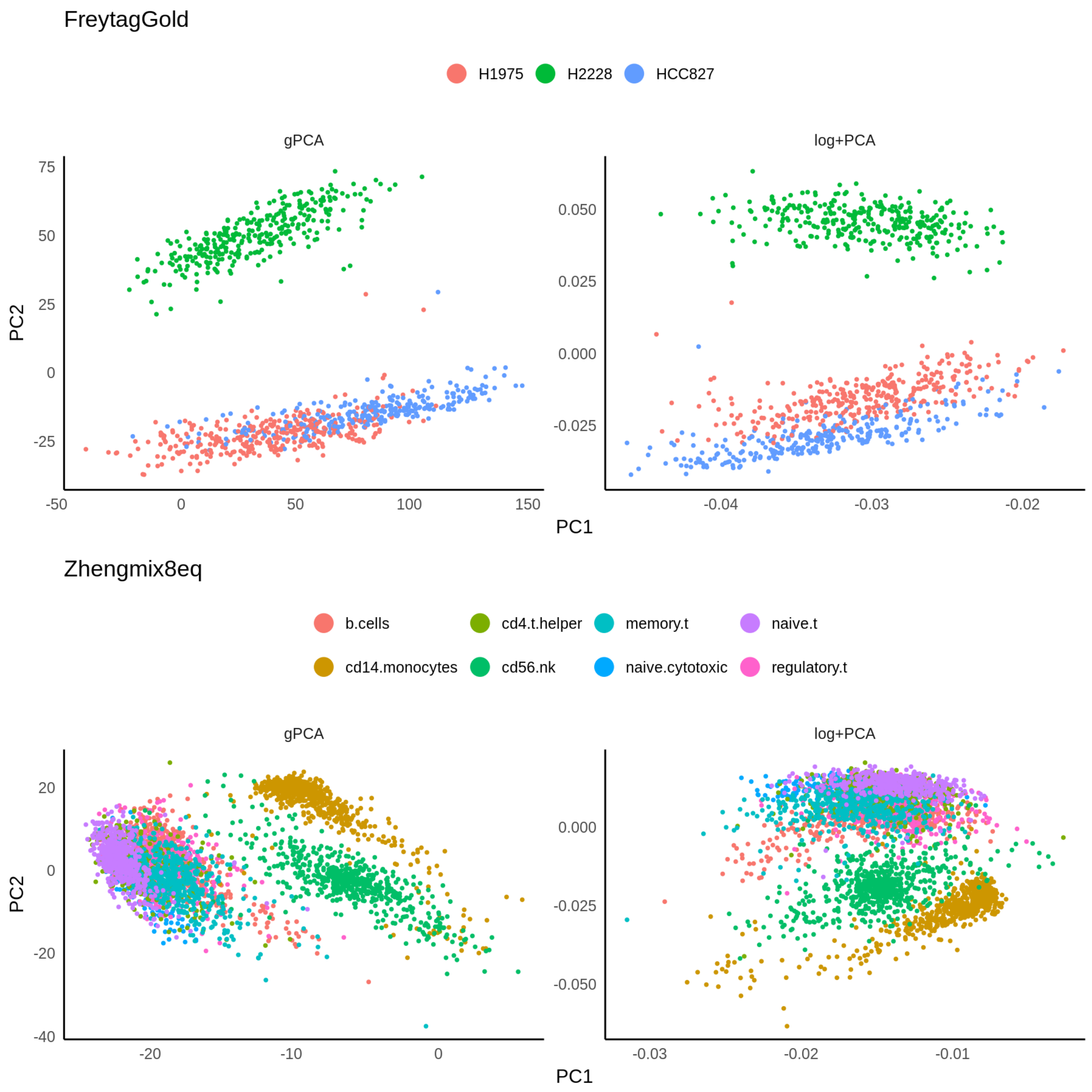

Figure S19: Generalized PCA (gPCA) [7] takes into acount the count structure of the data to reduce the dimensions, and could be integrated into HIPPO procedure. However, empirically, its results are similar to the result of log transformation + PCA, and the result does not make up for the computational burden of gPCA.

8 Analysis with HIPPO

8.1 Figure S20: HIPPO applied to Brain cells

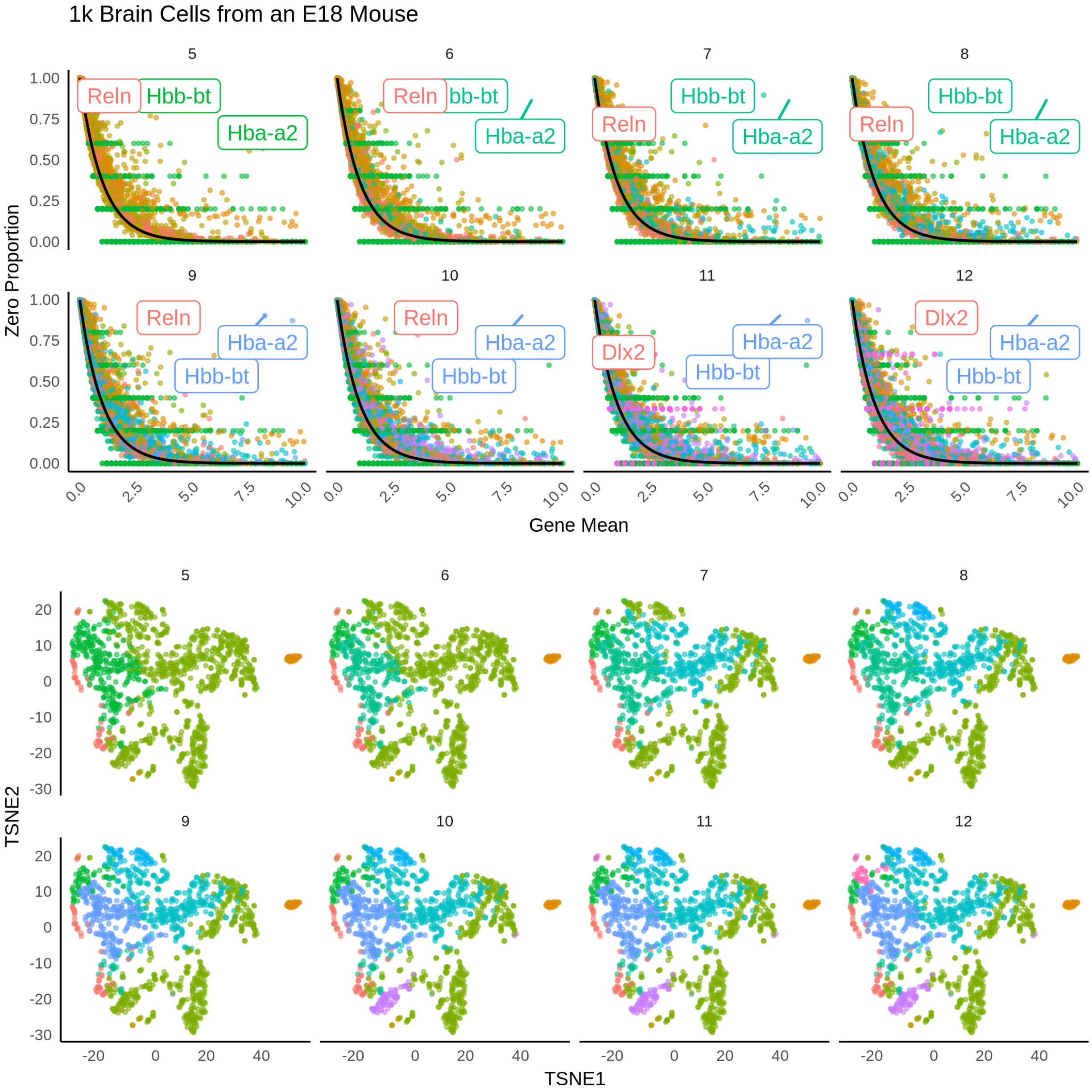

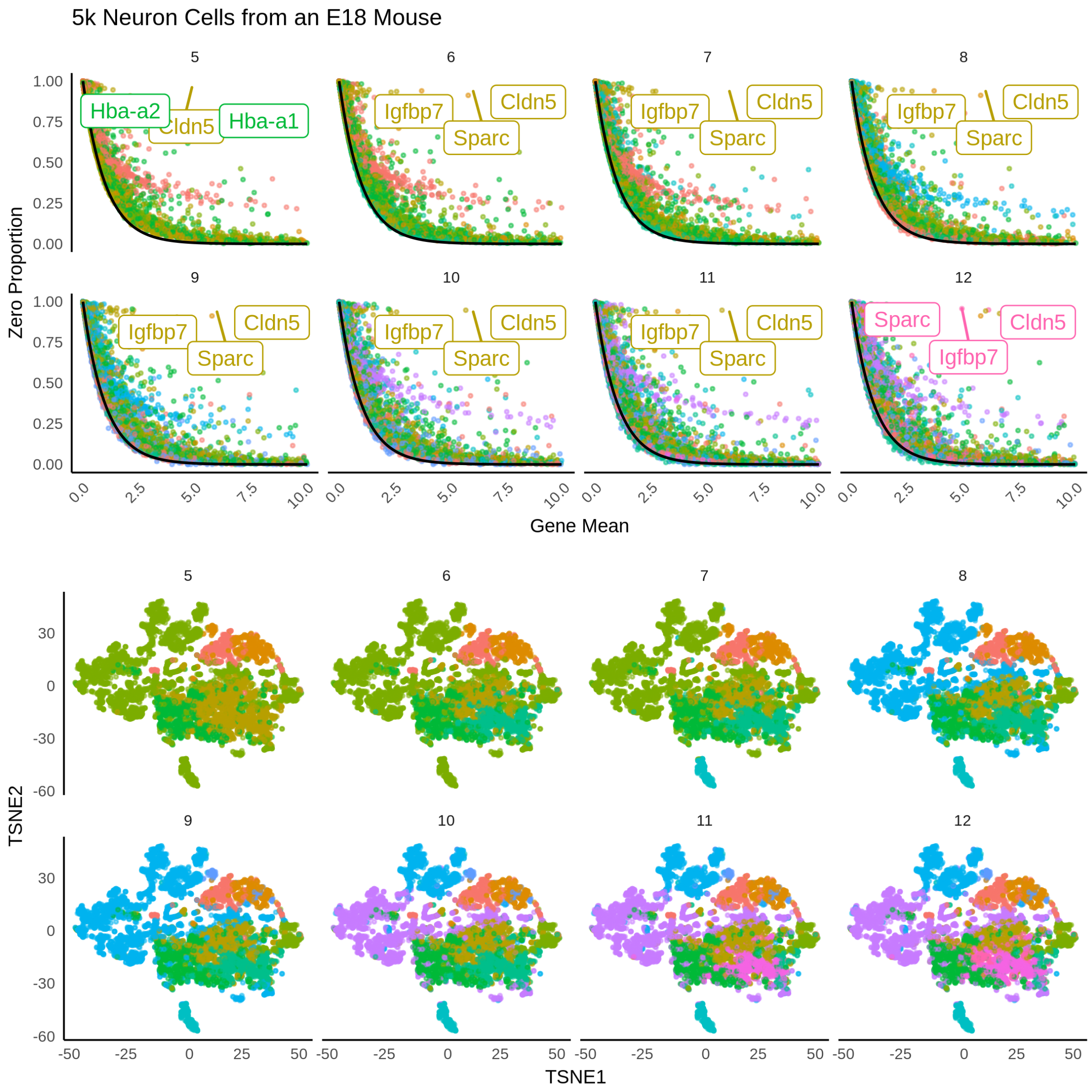

Figure S20: Example analysis of HIPPO for cells from two examples of brain tissues with higher number of clusters. For each round of clustering, zero proportions are more aligned to the Poisson line. The t-SNE plot is more finely separated as the number of clusters increase. HIPPO can show the differentiation of cell types in sequencing manner.

8.2 Figure S21: HIPPO Analysis pipeline

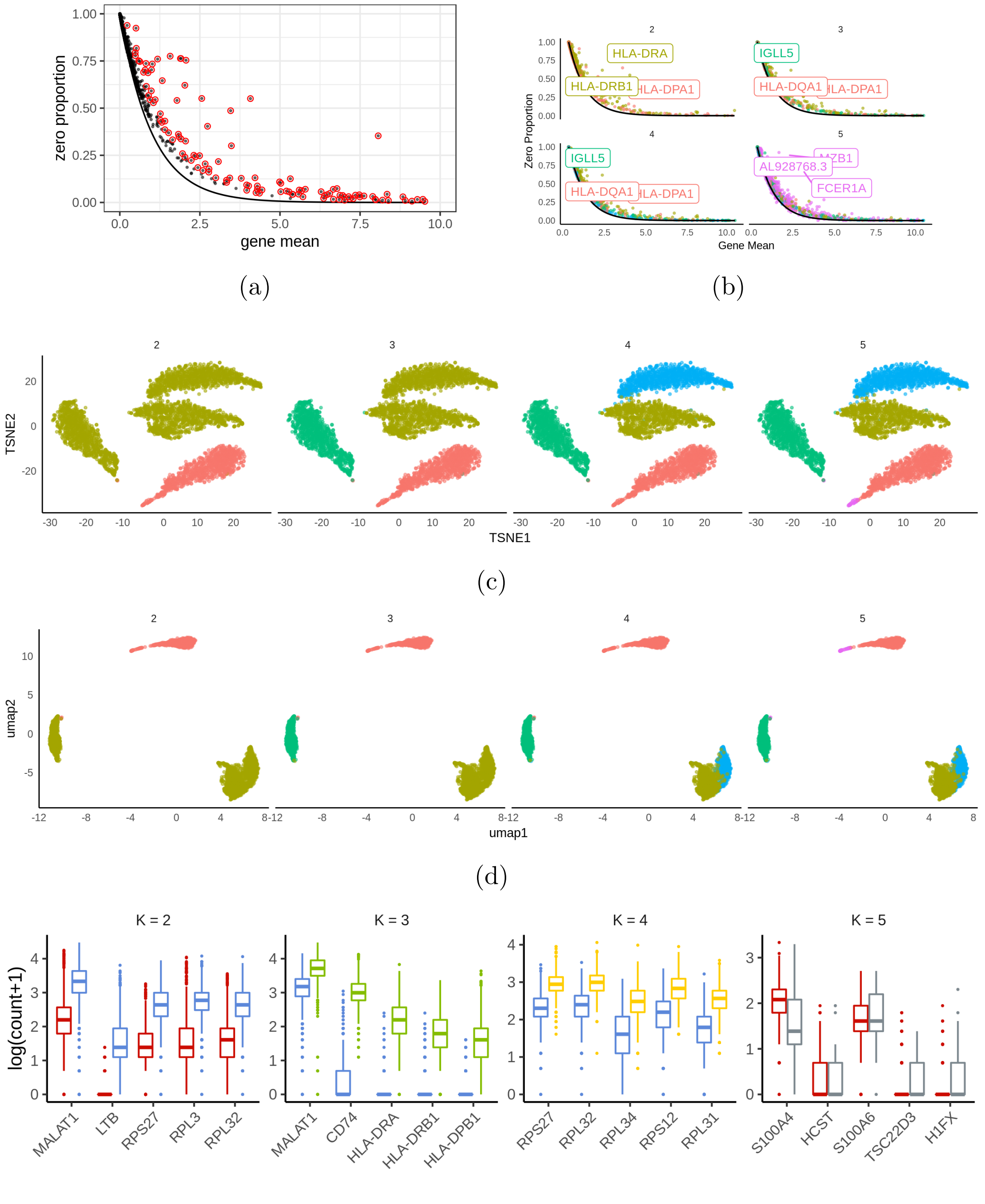

(a) (b) (c) (d) (e)

Figure S21: Sample analysis of Zhengmix4eq using HIPPO. The software first shows the diagnostic plot where zero-inflated genes are marked in red. Then it performs the clustering which leads to three sequential plots: zero proportions, t-SNE, and UMAP. Lastly, it shows the sequential differential expression analysis where color-coding matches the t-SNE and UMAP plots.

8.3 Figure S22: Two Methods of Differential Expression

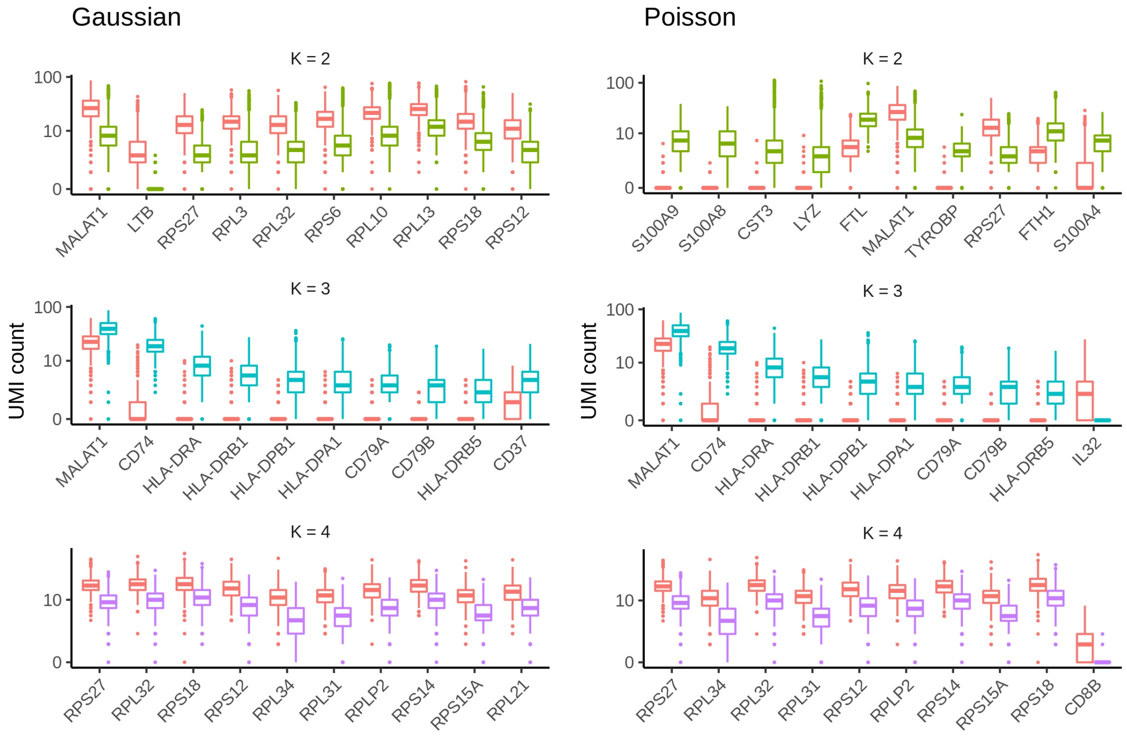

Figure S22: Example of two differential expression methods in Zhengmix4eq data. Results are very similar, and their test statistics' spearman correlations are 0.98, 0.99, and 0.99 respectively for $K = 2$, $K = 3$, and $K = 4$.

8.4 Figure S23: Tree structure of Hierarchical Clustering

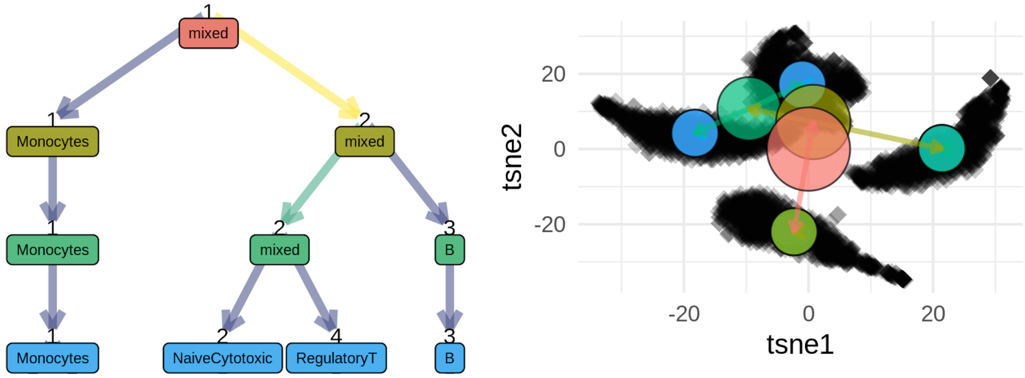

Figure S23: Clustree [13] package allows the tree-like visualization of the clustering result. The hierarchical structure gives insight to the overall structure of cell types and subtypes.

9 Text S1: Details about z-score

There are two ways to estimate the zero proportions. The observed zero proportion is $\hat{p}_g = \frac{\sum_{c=1}^{C} \mathbb{1}_{X_{gc}=0}}{C}$ which measures the proportion of cells with zero counts across all cells. The expected zero proportion is, under Poisson assumption, $e^{-\bar{X}_g}$ which is the exponential of the negative average count $\bar{X}_g$ which is used as a proxy for the true gene mean.

The distributions of the two estimates are as below.

- $\hat{p}_g \sim \mathcal{N}(0, \frac{p_g(1-p_g)}{C})$, meaning
$$E(\hat{p}_g) = p_g$$
$$Var(\hat{p}_g) = \frac{p_g(1-p_g)}{C}$$
- $e^{-\bar{X}_g} \sim log\mathcal{N}(\lambda_g, \lambda_g/C)$, which means
$$E(e^{-\bar{X}_g}) = p_g^{\frac{2C-1}{2C}}$$
$$Var(e^{-\bar{X}}) = (p^{-\frac{1}{C}} - 1)p^{\frac{2C-1}{C}}$$

The distribution of the difference of normal and log-normal distribution is not trivial, especially because $\hat{p}_g$ and $\bar{X}_g$ are not independent. For practical convenience, $e^{-\bar{X}_g}$ is assumed to be equal to $e^{-\lambda_g}$, the expected zero proportion using the unobserved, true gene mean.

One consequence of this method is that there is a small bias. $E(e^{-\bar{X}_g}) = p_g^{\frac{2C-1}{2C}}$ is greater than the true expected zero proportion $e^{-\lambda_g} = p_g$. However, as we deal with more than 1,000 cells, this difference is negligible and disappears asymptotically.

The second issue is the underestimation of variance — the variance of $e^{-\bar{X}}$ is ignored when the distribution of $\hat{p}_g - e^{-\bar{X}_g}$. However, the ultimate goal of this method is to select the features rather than making correct inferences. The z-score threshold is defined by the users, and they can alternatively choose to select top 2000 genes, in which case the variance does not have an impact on feature selection as the ordering of the important genes are not affected much by the variance. The method is still a valid approach for feature selection.

References

[1] Elham Azizi, Ambrose J Carr, George Plitas, Andrew E Cornish, Catherine Konopacki, Sandhya Prabhakaran, Juozas Nainys, Kenmin Wu, Vaidotas Kiseliovas, Manu Setty, et al. Single-cell map of diverse immune phenotypes in the breast tumor microenvironment. *Cell*, 174(5):1293–1308, 2018.

[2] Maayan Baron, Adrian Veres, Samuel L Wolock, Aubrey L Faust, Renaud Gaujoux, Amedeo Vetere, Jennifer Hyoje Ryu, Bridget K Wagner, Shai S Shen-Orr, Allon M Klein, et al. A single-cell transcriptomic map of the human and mouse pancreas reveals inter-and intra-cell population structure. *Cell systems*, 3(4):346–360, 2016.

[3] Tabula Muris Consortium et al. Single-cell transcriptomics of 20 mouse organs creates a tabula muris. *Nature*, 562(7727):367, 2018.

[4] Gökcen Eraslan, Lukas M Simon, Maria Mircea, Nikola S Mueller, and Fabian J Theis. Single-cell rna-seq denoising using a deep count autoencoder. *Nature communications*, 10(1):390, 2019.

[5] Saskia Freytag, Luyi Tian, Ingrid Lönnstedt, Milica Ng, and Melanie Bahlo. Comparison of clustering tools in r for medium-sized 10x genomics single-cell rna-sequencing data. *F1000Research*, 7, 2018.

[6] Mo Huang, Jingshu Wang, Eduardo Torre, Hannah Dueck, Sydney Shaffer, Roberto Bonasio, John I Murray, Arjun Raj, Mingyao Li, and Nancy R Zhang. Saver: gene expression recovery for single-cell rna sequencing. *Nature methods*, 15(7):539, 2018.

[7] Yoonkyung Lee. Generalized principal component analysis. *Journal of Educational Psychology*, 24(6):417–441, 2015.

[8] Evan Z Macosko, Anindita Basu, Rahul Satija, James Nemesh, Karthik Shekhar, Melissa Goldman, Itay Tirosh, Allison R Bialas, Nolan Kamitaki, Emily M Martersteck, et al. Highly parallel genome-wide expression profiling of individual cells using nanoliter droplets. *Cell*, 161(5):1202–1214, 2015.

[9] Mark D Robinson, Davis J McCarthy, and Gordon K Smyth. edger: a bioconductor package for differential expression analysis of digital gene expression data. *Bioinformatics*, 26(1):139–140, 2010.

[10] Luyi Tian, Xueyi Dong, Saskia Freytag, Kim-Anh Le Cao, Shian Su, Daniela Amann-Zalcenstein, Tom S Weber, Azadeh Seidi, Shalin Naik, and Matthew E Ritchie. scrna-seq mixology: towards better benchmarking of single cell rna-seq protocols and analysis methods. *BioRxiv*, page 433102, 2018.

[11] F. William Townes and Kelly Street. *scry: Small-Count Analysis Methods for High-Dimensional Data*, 2020. R package version 1.1.0.

[12] Po-Yuan Tung, John D Blischak, Chiaowen Joyce Hsiao, David A Knowles, Jonathan E Burnett, Jonathan K Pritchard, and Yoav Gilad. Batch effects and the effective design of single-cell gene expression studies. *Scientific reports*, 7:39921, 2017.

[13] Luke Zappia and Alicia Oshlack. Clustering trees: a visualization for evaluating clusterings at multiple resolutions. *GigaScience*, 7(7), jul 2018.

[14] Fan Zhang, Kevin Wei, Kamil Slowikowski, Chamith Y Fonseka, Deepak A Rao, Stephen Kelly, Susan M Goodman, Darren Tabechian, Laura B Hughes, Karen Salomon-Escoto, et al. Defining inflammatory cell states in rheumatoid arthritis joint synovial tissues by integrating single-cell transcriptomics and mass cytometry. *Nature immunology*, 20(7):928–942, 2019.

[15] Grace XY Zheng, Jessica M Terry, Phillip Belgrader, Paul Ryvkin, Zachary W Bent, Ryan Wilson, Solongo B Ziraldo, Tobias D Wheeler, Geoff P McDermott, Junjie Zhu, et al. Massively parallel digital transcriptional profiling of single cells. *Nature communications*, 8:14049, 2017.
