# Supplementary material for: Demystifying “drop-outs” in single-cell UMI data
Source: Genome Biol. 2020 Aug 6;21:196. doi: 10.1186/s13059-020-02096-y (PMC7412673; doi:10.1186/s13059-020-02096-y)
Supplement: Supplementary file 2 — Additional file 2 Review history. [file 13059_2020_2096_MOESM2_ESM.docx]

Review History

**First round of review**

**Reviewer 1**

**Were you able to assess all statistics in the manuscript, including the appropriateness of statistical tests used?**

Yes, and I have assessed the statistics in my report.

**Comments to author:**

This paper provides a comprehensive evaluation of distribution models of UMI count derived from scRNA-seq data from droplet-based technology. One important finding is that, once we consider each cell type separately, the UMI counts can be modeled by Poisson distributions. This is a much simpler model than many previous attempts in the field. Based on this observation, the authors proposed a simple strategy to perform feature selection. One key novelty of this feature selection method is that it only require computation of proportion of zero count, therefore alleviating the need for scaling of non-zero counts. Building on this approach, they developed a simple method to hierarchically cluster cells, and to perform differential expression analysis between two classes.

The level of innovation and significance of this paper is high. I suspect it will stimulate further methodological research in this area, including batch correction, trajectory analysis, etc.

One claim in page 5 (first sentence of the last paragraph) is that '...capture efficiencies in the 10X protocol affect UMI counts in different cell types and even across data sets in a similar fashion (Supplementary Figure 1,2)'. This is a very intriguing statement. In the Supplementary figures, indeed it is clear that the zero-UMI proportion is distributed more similarly between data sets of the same cell type than between different cell types of the same data set. Nonetheless, I wonder how we can assert that this is due the capture efficiency in the 10X protocol? Can the authors provide additional explanation or evidence? This is a very intriguing claim. If this is true, I wonder if we can identify a general cell-type specific 'lambda' (the Poisson parameter)? If this is possible, we may be able to use such a characteristic to identify cell type? Do the authors observe significantly different lambda across very different cell type? Is this related to different cell type characteristics (e.g., 'sticky' cells form solid tissue vs. blood cells)? This could be tested by looking at comprehensive tissue atlas such as Tabula Muris.

**Reviewer 2**

**Were you able to assess all statistics in the manuscript, including the appropriateness of statistical tests used?**

Yes, and I have assessed the statistics in my report.

**Comments to author:**

Review of Kim et al (2020) Genome Biology Article

=========

F. William Townes, Department of Computer Science, Princeton University

---------

\### Summary

Kim et al present HIPPO, a method for clustering single cell RNA-seq UMI counts without normalization. The contributions include a feature selection criterion based on zero-inflation and a clustering procedure based on iteratively reapplying feature selection and binary clustering which is compared to direct clustering on a single round of feature selection. They add some empirical support to the accumulation of recent studies arguing against zero-inflated models for UMI count data (ie, that a simple Poisson likelihood is probably sufficient). They also make the provocative claim that adjustment for the total count of each cell can be detrimental to identifying cell types. While many of the ideas are interesting, the supporting evidence is limited. I recommend the authors clarify their assumptions, compare against additional methods, and most importantly include datasets with a wider variety of protocols and cell types to buttress their conclusions.

\### Major concerns and questions

* The use of zero inflation as a feature selection criterion ignores the contribution of genes that have low but nonzero expression in one condition versus high expression in another. While I agree with the authors that the vast majority of information in low count UMI data are probably between the zeros and the nonzeros, I would like to see a more direct comparison to feature selection methods that use all of the information in the data. For example, we have recently released the scry bioconductor package to do feature selection based on the deviance of binomial and/or Poisson null models (https://bioconductor.org/packages/release/bioc/html/scry.html). I would be very curious to see if the results change when a deviance statistic is substituted for the zero inflation statistic in HIPPO. I expect there would be a strong correlation between the zero inflation statistic of each gene and its deviance statistic, such that either approach would give a similar result in downstream clustering performance. In addition, based on the results from Duo et al 2018 (https://f1000research.com/articles/7-1141/v2), feature selection simply by taking the genes with highest average expression should be included in the comparison as a baseline.

* When comparing different feature selection methods, it would be helpful for the authors to include a ground truth based on differentially expressed genes computed across the known cell types.

* p. 3 line 26: While I generally agree with the authors' claim that zero inflation is unnecessary to model UMI counts, an important counter-perspective is provided by Clivio et al 2019 (https://www.biorxiv.org/content/10.1101/794875v3). Please address their results in your revision.

* p. 4, lines 45-53: I found this part of the paragraph confusing. Why would we want to throw away genes that we know are heterogeneous just to reduce the granularity of a clustering? Why not do the clustering with all informative genes, then merge clusters post-hoc based on something like a stability criterion (https://jef.works/blog/2018/02/28/stability-testing/)?

* p. 5 lines 4-11 (also, Methods p. 16-17): as noted in the first pointer above, I am concerned that this test statistic does not use all the information in the data. From a statistical perspective, the authors are using a method of moments (MOM) estimator to characterize the entire distribution, whereas something like deviance is based on a maximum likelihood (MLE) framework. It is entirely possible that your MOM estimator has advantages over MLE approaches, such as in terms of robustness to model misspecification, but this may also come at a cost of reduced power etc. Please address this concern by showing under what conditions the MOM estimator breaks down (eg, small number of cells, negative binomial simulated data, highly expressed gene with not many zeros, etc). I would also like to see some simulations to see when the asymptotic approximations in Methods section "Feature Selection and Inference" break down. Finally, if the goal is to maintain robustness to modeling assumptions of the alternative hypothesis, why not do a score test? The score test only requires information from the null hypothesis. You could parameterize the Poisson by the zero probability instead of the mean, then obtain the score and the Fisher information according to this alternative parameter. Under the null hypothesis, the zero probability could equal exp(-mu) where mu is the sample mean, just as in your formulation. But the score statistic would have different statistical properties than your method of moments approach. I am very curious under what conditions each is advantageous.

* p. 5 line 13: I am confused why this binomial assumption is needed. Do you mean a Bernoulli distribution with p(y=0) given by exp(-lambda_g)? If not, what is the "N" in the binomial? I could not find any reference to binomial distributions in the corresponding methods subsection, which appears to instead be based on a lognormal distribution.

* p. 6 first paragraph/ figure 2: the claim that total UMI counts are biologically informative is very interesting and provocative. Like Hafemeister & Satija (2019), in our work we have "adjusted for" total UMI counts (referred to by the authors as "sequencing depth") because we believe them to be completely confounded with technical variation. Thus, without ground truth cell type labels there is no way to be sure whether differences in cells due to total UMI are biological or technical. Of course, if we assume the technical processing of each cell in a dataset is very consistent and uniform, then yes we acknowledge the authors' claim that eg larger cells can be expected to have larger total UMI count. However, the comparisons shown are not very convincing since they are limited to the 10x protocol (which chemistry?), and seem to only examine B cells vs monocytes. The authors should either narrow their claim to reflect the limited comparisons, or include more comparisons of different cell types and protocols. For example, they could incorporate the CEL-seq2 data from Zhang et al 2019 (https://www.nature.com/articles/s41590-019-0378-1 , see also attached Rmd file) which contains B-cells and monocytes as well as T-cells and fibroblasts, but the total UMI counts for all cell types are much higher than in the comparable 10x Zheng PBMC dataset. So, in an updated Figure 2 it would be nice to see not just B-cells vs monocytes but also fibroblasts and various types of T-cells. My question is, if you concatenate the Zhang and Zheng datasets, are the biggest differences across cells in terms of total UMI coming from the cell types or the protocol? I suspect it will be the latter. If so, the authors should really emphasize that the informativeness of total counts to identify cell types is heavily dependent on the assumption of uniform processing (ie no batch effects or differences in protocols across cells). If they want to be able to make a broader claim, they may want to allow for covariate adjustment in computing the zero inflation statistic. For example, if you have cells from multiple batches where total UMI varies a lot between batches and confounds cell type identification, you could just have a categorical batch label covariate and remove this confounder when computing the test statistic, to avoid just finding genes that vary a lot across batches. In addition to the Zhang 2019 dataset, I would recommend exploring the Tung et al 2017 dataset (https://www.nature.com/articles/srep39921?report=reader, see also attached Rmd file) as a negative control. All the cells in this dataset are the same type, but they come from multiple replicates from different individuals, and the total UMI counts are highly variable across these batches.

* p. 7: HIPPO- the idea of iterative clustering and feature selection is a good one, but there is no reason why users of your tool should be restricted to a particular dimension reduction like PCA and/or clustering algorithm like K-means. I suggest you make the implementation modular so that users can provide any dimension reduction or clustering function of their choice. It would be helpful if you could also illustrate HIPPO with a more powerful clustering method such as Louvain or Leiden. I don't think the results will change much but it would make your results more convincing and increase the number of people interested in the method.

* p. 17, lines 25-30 (Methods): please provide an explicit formula for the Z-statistic. I think it would just be a rearrangement of terms in the last equation.

* p. 17 (Methods: "Hierarchical Clustering"): A more specific description of the procedure needs to be provided. Please give advice to users on a good stopping procedure. Does intra-cluster distance adjust for number of cells? Is it relative to inter-cluster distance or just an absolute number? Have you considered using something more standard like silhouette coefficient as a criterion? What transform (if any) is used as input to PCA (eg log2(1+counts)?). Centering and scaling by feature? How many PCs/ dimensions used as input to clustering? Was Euclidean distance to cluster centers computed in the PCA reduced space or the original data space?

* p. 18 (Methods: "Differential Expression Testing"): the t-test assumes a Gaussian likelihood which contradicts the message of the rest of the paper. Why not do a proper Poisson GLM to compare the two groups? I imagine the Wald statistic and p-values could be obtained in closed form.

\### Minor comments and suggestions

* p. 2 line 41 "adversary" should be changed to "adverse".

* You may want to cite a recent benchmarking paper from Germain et al 2020 (https://www.biorxiv.org/content/10.1101/2020.02.02.930578v2) in the introduction.

* In the last paragraph of the intro, it might be worth citing Sarkar & Stephens 2020 (https://www.biorxiv.org/content/10.1101/2020.04.07.030007v2.abstract). They further support your claim about the Poisson being good enough as a noise model.

* p. 3 line 27: "standard normal distribution" should be changed to "asymptotic standard normal distribution under the null hypothesis".

* p. 3 line 57: "sequencing depth" should probably be changed to "total UMI count".

* p. 4, line 27: "protein-coding genes" should probably be changed to "non-immune genes".

* p. 5, line 18: you may want to use the Holm correction for multiple testing since it is uniformly more powerful than the Bonferroni adjustment (https://www.jstor.org/stable/4615733) see also the documentation for p.adjust in R, where Holm is the default.

* p. 5, line 34: "sufficient for cellular heterogeneity analysis" is a broad claim, I suggest this be modified to emphasize the method is intended for low counts. Now that smart-seq3 is out, I suspect there will be more UMI datasets with large counts, where zero-inflation tests could be less powerful.

* p. 6, line 50: "accounts" should be "counts"

* p. 7, line 50: thank you for not hiding this scenario where the method breaks down, I appreciate your honesty!

* Table 2- should probably mention the chemistry version for the 10x datasets (eg, chromium v1/v2/v3 or gemcode). May also want to add a column saying the tissue type of each dataset, it can be guessed from second column for some, but not clear for all.

* Both the Hafemeister and our (Townes) papers have been published in Genome Biology, so you may want to update the citation (currently it cites biorxiv).

* In your github readme file, you describe installing the Zhengmix4eq dataset from a URL. It may be easier to just install it from the DuoClustering2018 bioconductor package (https://www.bioconductor.org/packages/release/data/experiment/html/DuoClustering2018.html ). This would also give you quick access to their filtered versions of the data based on highly variable genes, average expression, M3Drop, etc.

\### Attachments

R markdown (.Rmd) files with instructions for loading the Zhang 2019 and Tung 2017 datasets, perhaps this will save you some time to avoid having to re-do all the preprocessing from scratch.

**Authors Response**

**Point-by-point responses to the reviewers’ comments:**

Responses to Reviewer #1

Reviewer #1: This paper provides a comprehensive evaluation of distribution models of UMI count derived from scRNA-seq data from droplet-based technology. One important finding is that, once we consider each cell type separately, the UMI counts can be modeled by Poisson distributions. This is a much simpler model than many previous attempts in the field. Based on this observation, the authors proposed a simple strategy to perform feature selection. One key novelty of this feature selection method is that it only requires computation of proportion of zero count, therefore alleviating the need for scaling of non-zero counts. Building on this approach, they developed a simple method to hierarchically cluster cells, and to perform differential expression analysis between two classes.

The level of innovation and significance of this paper is high. I suspect it will stimulate further methodological research in this area, including batch correction, trajectory analysis, etc.

*Thank you for your encouraging words!*

One claim in page 5 (first sentence of the last paragraph) is that '...capture efficiencies in the 10X protocol affect UMI counts in different cell types and even across data sets in a similar fashion (Supplementary Figure 1,2)'. This is a very intriguing statement. In the Supplementary figures, indeed it is clear that the zero-UMI proportion is distributed more similarly between data sets of the same cell type than between different cell types of the same data set. Nonetheless, I wonder how we can assert that this is due the capture efficiency in the 10X protocol? Can the authors provide additional explanation or evidence? This is a very intriguing claim.

*Thank you for the comment. We changed the statement to “… the droplet-based data-generating process in the 10X protocol affect UMI counts …” as it is difficult to prove, as you suggest, that such phenomenon is driven by the capture efficiency solely.*

If this is true, I wonder if we can identify a general cell-type specific 'lambda' (the Poisson parameter)? If this is possible, we may be able to use such a characteristic to identify cell type? Do the authors observe significantly different lambda across very different cell type?

*Thank you for the comment. We wonder if there has been miscommunication with Supplementary Figures 1 and 2. All Poisson curves in the figures are simple function e^{-lambda} where x-axis is the lambda parameter and y-axis, for each lambda value, is P(X=0) when X ~ Poisson(lambda). Thus, these Poisson curves are not characterized by cell-type specific parameters. We claim that each Poisson curve is sufficient to describe the drop-out vs mean pattern across all genes, across all cell-types, across all data sets. Having made the previous points clear, gene means (lambda) are not comparable across data sets because they may be influenced by batch effect. Lambdas after appropriate batch effects correction might be used more reliably to identify cell types. We have added assumptions in the manuscript that our conclusions are based on the UMI data sequenced in one batch.*

*Inspired by your comment, we conducted the following analysis. We two batches of SRP073767 – PBMC3K from 10X GemCode and PBMC4K from 10X Chromium – and computed the gene means of different cell types. We plotted all genes for 11 cell types, where x-axis is the estimated gene mean (average UMI count) from PBMC3K data and y-axis is from PBMC4K. The gene means are very highly correlated even without correcting for batch effects, except one cell type of CD34+. We believe this is due to the internal heterogeneity explained in Figure 1. This analysis provides an optimistic view of comparing lambdas across batches, but we would not recommend it as of now.*

*Figure 1. Comparison of two batches in the same data (SRP073767) by estimating gene mean by averaging the UMI counts. Most cell-types align well except CD34+, known to have internal heterogeneity (Main text Figure 1 C), and Dendritic cells.*

*Figure 2. Heatmap of Pearson correlations between gene means for each batch and each cell-type. Monocytes and NK cells clearly show that gene means are highly correlated across different batches compared to other cell types. T cells are in general all highly correlated, and some feature selection is necessary to differentiate more granular cell types.*

Is this related to different cell type characteristics (e.g., 'sticky' cells form solid tissue vs. blood cells)? This could be tested by looking at comprehensive tissue atlas such as Tabula Muris.

*Thank you for the suggestion and reference to the data set. Unfortunately, the lambdas are difficult to compare among data sets across different batches. We would need controlled experiment where batch effects are all corrected out in order to compare gene means estimated by the average UMI count.*

*Still, we added extra analysis from Tabula Muris data to further support our claim. For a given cell types that are present in more than 4 tissues, namely macrophage, T cell, endothelial cell, and leukocyte, the zero proportions follow Poisson distribution very closely across different tissues. But, again, since each tissue was sequenced separately in different batches, it is difficult to make meaningful conclusions about gene mean comparison across different tissues.*

*Figure 3 (Supplementary Figure 3). Analysis of Tabula Muris for 4 cell types that sequenced for more than 4 tissues. Regardless of cell types or tissues, the zero proportions align well with the expected Poisson curve with some exceptions that show mild heterogeneity (endothelial cells and macrophage).*

Reviewer #2: Review of Kim et al (2020) Genome Biology Article

=========

F. William Townes, Department of Computer Science, Princeton University

---------

\### Summary

Kim et al present HIPPO, a method for clustering single cell RNA-seq UMI counts without normalization. The contributions include a feature selection criterion based on zero-inflation and a clustering procedure based on iteratively reapplying feature selection and binary clustering which is compared to direct clustering on a single round of feature selection. They add some empirical support to the accumulation of recent studies arguing against zero-inflated models for UMI count data (ie, that a simple Poisson likelihood is probably sufficient). They also make the provocative claim that adjustment for the total count of each cell can be detrimental to identifying cell types. While many of the ideas are interesting, the supporting evidence is limited. I recommend the authors clarify their assumptions, compare against additional methods, and most importantly include datasets with a wider variety of protocols and cell types to buttress their conclusions.

*Thank you for your comment. We appreciate that you have brought important concerns, and to respond, we have (1) conducted additional analyses with more data sets from different tissues and protocols (Tung et al., Zhang et al., Tabula Muris) and (2) made clear the scope of our claims throughout the manuscript. Details are below.*

\# Major concerns and questions

* The use of zero inflation as a feature selection criterion ignores the contribution of genes that have low but nonzero expression in one condition versus high expression in another. While I agree with the authors that the vast majority of information in low count UMI data are probably between the zeros and the nonzeros, I would like to see a more direct comparison to feature selection methods that use all of the information in the data. For example, we have recently released the scry bioconductor package to do feature selection based on the deviance of binomial and/or Poisson null models (https://bioconductor.org/packages/release/bioc/html/scry.html). I would be very curious to see if the results change when a deviance statistic is substituted for the zero inflation statistic in HIPPO. I expect there would be a strong correlation between the zero inflation statistic of each gene and its deviance statistic, such that either approach would give a similar result in downstream clustering performance. In addition, based on the results from Duo et al 2018 (https://f1000research.com/articles/7-1141/v2), feature selection simply by taking the genes with highest average expression should be included in the comparison as a baseline.

* When comparing different feature selection methods, it would be helpful for the authors to include a ground truth based on differentially expressed genes computed across the known cell types.

*We appreciate your comment and understand your concern. The comparison of feature selection method is illustrated in Figure 2 F, where we compare the features selected using the likelihood ratio statistic (that tests between Poisson vs NB) and using the zero-inflation statistic. The blue line shows the distribution of features’ gene means that were both selected by the two methods.*

*Following your comments, we have included further analyses in the Supplementary Materials where we compare the two packages “scry” and “HIPPO”. Specifically, we compared the relationship between those statistics and the mean counts of each gene.*

*Figure 4 (Supplementary Figure 9). Comparison of the test statistics between the proposed package HIPPO and package scry for two data sets Freytag2018 and Zheng2017. Both x-axis and y-axis are log-scaled. The ordering of the statistic is similar between zero inflation test statistic and deviance statistic, although the zero-inflation test does not take into account the entire distribution of the gene counts. There are a few genes that have high deviance but low zero inflation in Freytag data. Those cases occur when there are no zeros recorded across all cells. Then, zero-inflation test statistic becomes lower as gene mean increases.*

*In most cases, both statistics have positive relationship with the gene mean with some notable exceptions. In Freytag2018 data, there are a few genes with very low zero inflation statistics with high gene mean. These scenarios occur when there are no zeros observed at all across all cells, and they are very rare when we deal with low-count UMI data sets.*

*A more extreme case is shown below. Tian 2018 data has much higher sequencing depth, and there are many genes that have very low zero inflation test statistics and high mean counts. Regardless, HIPPO successfully clusters the cell lines in this data set by just using those genes that have at least one cell recorded 0. Therefore, empirically, we still believe zero inflation test is a robust measure for feature selection.*

*Figure 5 (Parts of Supplementary Figure 10). Same analysis as Supplementary Figure 8 with Tian2018 data which has higher UMI counts. This analysis shows different relationship between zero inflation test and deviance test. When the mean counts become large, the zero-inflation test statistic is either extremely low (there are no zeros recorded) or extremely high (there is at least 1 zero recorded). The problem is more severe when there are fewer cells as arguments for the test statistic are asymptotic. However, the HIPPO result shows that the zero-inflated genes still hold rich information for reliable clustering.*

*We added the reservation that the zero-inflation test is more appropriate for low-UMI-count data sets in the discussion section in page 9: “Thirdly, the zero inflation statistics are appropriate for data sets with relatively low sequencing depth. As an extreme example, when all gene counts are high and there are no or very few cells with zero counts, zero-inflation test is not valid.” Moreover, we added a new function in HIPPO software so that users can choose whether to use deviance or zero-inflation as their feature selection method. Below, we compare the clustering performance of the two feature selection methods within the HIPPO framework. The results are dependent on the test statistic thresholds (deviance threshold and zero-inflation threshold), but we conclude that the clustering performances are similar.*

*Figure 6-1( Parts of(Supplementary Figure 11). Clustering performance comparison for two feature selection method for Tian2018 data (high UMI count).*

*Figure 6-2 (Supplementary Figure 12). Clustering performance comparison between two feature selection for Zhengmix4uneq (low UMI count). The truth labels are shown for both ways of dimension reduction. Overall, their clustering performances are very similar.*

*Here, we would like to set our scope of study more clearly. By doing the zero-inflation test, we are not claiming the highly expressed genes to be ‘unimportant.’ But we observe that the zeros in many UMI data sets have rich information for effective clustering. Empirically, examining zeros without looking at highly expressed genes is sufficient for clustering.*

* p. 3 line 26: While I generally agree with the authors' claim that zero inflation is unnecessary to model UMI counts, an important counter-perspective is provided by Clivio et al 2019 (https://www.biorxiv.org/content/10.1101/794875v3). Please address their results in your revision.

*Thank you for the reference. Clivio et al 2019’s analysis shows that, in 10X data, “higher fraction of genes are zero-inflated within gene-cell-type than control RNAs.” However, this observation does not contradict our claim that zero-inflation is an indicator of cellular heterogeneity. We admit in Section 1 that different sets of genes have different granularity of cell groupings. For example, even within one cell type, immune-related genes tend to be more heterogeneous and hence to be more zero-inflated. We have cited their results at the end of page 4 by more clearly explaining what it means to expect varying levels of biological heterogeneity among genes.*

* p. 4, lines 45-53: I found this part of the paragraph confusing. Why would we want to throw away genes that we know are heterogeneous just to reduce the granularity of a clustering? Why not do the clustering with all informative genes, then merge clusters post-hoc based on something like a stability criterion (https://jef.works/blog/2018/02/28/stability-testing/)?

*Thank you for pointing out the mistake in the manuscript. We do not exclude the immune genes when we select the features or do the clustering. We have re-phrased the part to accurately explain what our method is doing: “The high level of cell heterogeneity in certain genes suggests that it is difficult, to say the least, to fully account for the cell type for every single gene; as we finely cluster the cells, we will reach a point where there are less than enough number of cells in each cluster to do statistically meaningful analysis. Still, it is practically important to resolve the cell type heterogeneity even with lower granularity, in which case we use a criterion for the stopping point of iterative clustering: the iterative procedure stops when there are less than a certain number of genes that are zero-inflated.”*

*The main point here is that there are certain genes with much higher heterogeneity than others due to their biology, such as IG C genes and TR C genes as illustrated in the manuscript. We could expect to observe such higher heterogeneity in future data sets.*

*Creating a “blacklist” of genes require more research and evidence, and we agree that immune genes hold valuable information about the cell because of their heterogeneity themselves. We instead use this information to provide a guideline for the stopping criterion of clustering. After we hit a certain low number of genes that pass the zero-inflation test, we can stop the clustering because our goal is not to “completely” resolve the cell heterogeneity.*

* p. 5 lines 4-11 (also, Methods p. 16-17): as noted in the first pointer above, I am concerned that this test statistic does not use all the information in the data. From a statistical perspective, the authors are using a method of moments (MOM) estimator to characterize the entire distribution, whereas something like deviance is based on a maximum likelihood (MLE) framework. It is entirely possible that your MOM estimator has advantages over MLE approaches, such as in terms of robustness to model misspecification, but this may also come at a cost of reduced power etc. Please address this concern by showing under what conditions the MOM estimator breaks down (eg, small number of cells, negative binomial simulated data, highly expressed gene with not many zeros, etc). I would also like to see some simulations to see when the asymptotic approximations in Methods section "Feature Selection and Inference" break down. Finally, if the goal is to maintain robustness to modeling assumptions of the alternative hypothesis, why not do a score test? The score test only requires information from the null hypothesis. You could parameterize the Poisson by the zero probability instead of the mean, then obtain the score and the Fisher information according to this alternative parameter. Under the null hypothesis, the zero probability could equal exp(-mu) where mu is the sample mean, just as in your formulation. But the score statistic would have different statistical properties than your method of moments approach. I am very curious under what conditions each is advantageous.

*Thank you for your comment. Score test is a great suggestion, but we decided that it is beyond the scope of this paper because it is asymptotically identical to the likelihood ratio test. Since our main concern is data sets with large sample size, the score test would be expected to yield very similar results to the likelihood ratio test.*

*In addition to the analysis results that compare different feature selection methods (Seurat, etc. in Figure 2 and scry in Supplementary Figure 9 and 10), as suggested in your first comment, we ran simulations to compare likelihood ratio tests using MLE and zero-inflation test using MOM (Supplementary Figure 13).*

*We generated count data in two alternative models: Poisson mixture (with lambdas 2 and 5 with mixture proportions 0.2 and 0.8) and Negative binomial (size 10 and prob 0.8 as parametrized in rnbinom function in R). The null hypothesis is that they come from Poisson distribution. We used mixtools package in R to use EM algorithm to estimate the Poisson mixture.*

*When the model was correctly specified, i.e. when data was generated from Poisson mixture, MLE was more powerful than the proposed ZI test. However, when data was generated from Negative Binomial, the estimation tool became highly unreliable and showed poor convergence behavior (Figure 6). Another drawback of MLE is its computational burden. EM algorithm takes longer than the proposed ZI test in the order of 10^4 to 10^5 (Figure 7).*

*We would like to note that statistical power is not of our greatest interest, because feature selection can be as liberal and as conservative as one user wants. The p-values, which are used to determine statistical power, are not very interpretable in the context of feature selection (although they are more important in differential expression analysis).*

*Figure 6 (Supplementary Figure 13). Comparison of Poisson Mixture MLE and proposed ZI test. When data was generated from Negative Binomial, MLE is unreliable. When model is correctly specified, statistical power is greater in MLE.*

*Figure 7 (Supplementary Figure 13). Computational Time comparison between MLE and proposed zero inflation test.*

*Our conclusions are not affected. Our main claim is that resolving the cellular heterogeneity should be the first step of the analysis pipeline. Our second claim is that zero inflation, rather than being “technical drop-outs”, holds valuable biological information about the cellular heterogeneity. In the process of our investigation of the biological signals of zero inflation, the methodology has been developed. And we are not claiming the superiority of zero inflation test over other feature selection methods excepts its computational and conceptual simplicity.*

* p. 5 line 13: I am confused why this binomial assumption is needed. Do you mean a Bernoulli distribution with p(y=0) given by exp(-lambda_g)? If not, what is the "N" in the binomial? I could not find any reference to binomial distributions in the corresponding methods subsection, which appears to instead be based on a lognormal distribution.

*Thank you for pointing out the mistake. Indeed, in this particular context, Bernoulli is the correct distribution to use. Only a Bernoulli assumption is required. We use N to denote the number of cells. We have made the correction.*

* p. 6 first paragraph/ figure 2: the claim that total UMI counts are biologically informative is very interesting and provocative. Like Hafemeister & Satija (2019), in our work we have "adjusted for" total UMI counts (referred to by the authors as "sequencing depth") because we believe them to be completely confounded with technical variation. Thus, without ground truth cell type labels there is no way to be sure whether differences in cells due to total UMI are biological or technical. Of course, if we assume the technical processing of each cell in a dataset is very consistent and uniform, then yes we acknowledge the authors' claim that eg larger cells can be expected to have larger total UMI count. However, the comparisons shown are not very convincing since they are limited to the 10x protocol (which chemistry?), and seem to only examine B cells vs monocytes. The authors should either narrow their claim to reflect the limited comparisons, or include more comparisons of different cell types and protocols. For example, they could incorporate the CEL-seq2 data from Zhang et al 2019 (https://www.nature.com/articles/s41590-019-0378-1 , see also attached Rmd file) which contains B-cells and monocytes as well as T-cells and fibroblasts, but the total UMI counts for all cell types are much higher than in the comparable 10x Zheng PBMC dataset. So, in an updated Figure 2 it would be nice to see not just B-cells vs monocytes but also fibroblasts and various types of T-cells. My question is, if you concatenate the Zhang and Zheng datasets, are the biggest differences across cells in terms of total UMI coming from the cell types or the protocol? I suspect it will be the latter. If so, the authors should really emphasize that the informativeness of total counts to identify cell types is heavily dependent on the assumption of uniform processing (ie no batch effects or differences in protocols across cells). If they want to be able to make a broader claim, they may want to allow for covariate adjustment in computing the zero inflation statistic. For example, if you have cells from multiple batches where total UMI varies a lot between batches and confounds cell type identification, you could just have a categorical batch label covariate and remove this confounder when computing the test statistic, to avoid just finding genes that vary a lot across batches. In addition to the Zhang 2019 dataset, I would recommend exploring the Tung et al 2017 dataset (https://www.nature.com/articles/srep39921?report=reader, see also attached Rmd file) as a negative control. All the cells in this dataset are the same type, but they come from multiple replicates from different individuals, and the total UMI counts are highly variable across these batches.

*Thank you for your comment. We agree that further assumption is necessary to claim the informativeness of total UMI count, and we indeed implicitly assumed that all cells are processed in one batch using one protocol.*

*First of all, we added the chemistry version for the 10X protocol in Table 2 and Supplementary Table 1. Some papers did not clearly mention the version number, and we added as much details as we could.*

*We repeated the same analysis with all four types included in Zhang data set (thank you for the markdown file!) In this data, there was no drastic difference between B cells and monocytes in their total UMI counts. Please look at the box plot below.*

*Figure 9. Distribution of total UMI counts for each cell type. While B cells, Monocytes, and T cells show similar centers of distribution, Fibroblasts show higher UMI counts than the rest. Forcing all cell types to have the same UMI counts through normalization could reduce the signals from biological markers of fibroblasts*

*However, we do not believe that our results can expand to the CEL-seq2 data sets. In Supplementary Figure 5, cell types all seem zero-inflated, and more importantly, cells from different disease types show a layered pattern of zero inflation. This means that our Poisson noise model cannot separate the technical effect from the biological confounder of disease type. We wouldn’t claim that our conclusion about the informativeness of total UMI count expands to the CEL-seq2 data sets.*

*Figure 10 (Supplementary Figure 5). When the cells are divided into (1) two diseases and (2) cell types, all genes are consistently zero-inflated compared to the expected Poisson curve.*

*Regardless, we would like the readers to consider the idea that total UMI count contains biological information such as cell size, as your comment mentioned.*

*In page 6, we have mapped out our assumption clearly that total UMI counts can represent technical factor when multiple data sets need to be integrated: “Total UMI count needs to be carefully corrected in case of data integration. Data sets collected from different protocols and batches have different distribution of UMI counts. However, when there is no batch effect, the total UMI count has valuable biological information.”*

*Furthermore, we limit our claim to say that B cells having more UMI counts than monocytes might not hold in different organisms or different tissues: “The UMI counts for different cell types do not need to be consistent across different tissues or organisms.” (page 6)*

* p. 7: HIPPO- the idea of iterative clustering and feature selection is a good one, but there is no reason why users of your tool should be restricted to a particular dimension reduction like PCA and/or clustering algorithm like K-means. I suggest you make the implementation modular so that users can provide any dimension reduction or clustering function of their choice. It would be helpful if you could also illustrate HIPPO with a more powerful clustering method such as Louvain or Leiden. I don't think the results will change much but it would make your results more convincing and increase the number of people interested in the method.

*Thank you for the comment.*

*The software is constantly evolving with added functionalities. We have added your concern to the “issue” page in github, and we will try to incorporate more powerful, different clustering methods in the software. However, one main advantage of HIPPO is that it is computationally fast (Figure 3 E-F ). Our choice of PCA + KMeans is meant to show that such simple clustering method can work “just as well” in some cases. Thank you for your suggestion.*

* p. 17, lines 25-30 (Methods): please provide an explicit formula for the Z-statistic. I think it would just be a rearrangement of terms in the last equation.

*Thank you for your comment. The z-statistic is now explicitly written as a formula.*

* p. 17 (Methods: "Hierarchical Clustering"): A more specific description of the procedure needs to be provided. Please give advice to users on a good stopping procedure. Does intra-cluster distance adjust for number of cells? Is it relative to inter-cluster distance or just an absolute number? Have you considered using something more standard like silhouette coefficient as a criterion? What transform (if any) is used as input to PCA (eg log2(1+counts)?). Centering and scaling by feature? How many PCs/ dimensions used as input to clustering? Was Euclidean distance to cluster centers computed in the PCA reduced space or the original data space?

*We appreciate the detailed comment. The hierarchical clustering is now explained more in detail including the points that you have addressed.*

*“Meanwhile, during the hierarchical clustering, HIPPO keeps track of intra-cluster variation by performing un-scaled, un-centered PCA. HIPPO computes the first 10 dimensions (user-defined) of cell embeddings and sum up the sample variance of each component. The PCA for recording the intra-cluster variability is not scaled to avoid potential bias due to the cell population size. Since a subset of cells are considered for clustering at each round, fewer and fewer cells are used for the dimension reduction. If scaled, their dimension-reduced cell embeddings would be artificially more far apart compared to when more cells were considered for clustering. The intra-cluster variance is the criterion for selecting the cell group to be further clustered in the next round.”*

*More details have been added to the summarized Algorithm 1 below.*

* p. 18 (Methods: "Differential Expression Testing"): the t-test assumes a Gaussian likelihood which contradicts the message of the rest of the paper. Why not do a proper Poisson GLM to compare the two groups? I imagine the Wald statistic and p-values could be obtained in closed form.

*Thank you very much for your comment. We agree that it is more consistent to use Poisson GLM for our differential expression analysis, and we have updated our method in HIPPO software. We would like to note that we are not assuming the Gaussian likelihood on the count data by doing the t-test. We are testing whether the two groups have the same mean, where the average counts asymptotically follow normal distribution by the Central Limit Theorem. We edited the software so that users can choose whether to use Gaussian approximation or Poisson likelihood ratio test. Figure 9 below is one example of the different top genes selected using the two different methods, and the rankings of the test statistics are very highly correlated (spearman correlation > 0.97 in all cases tested).*

*Figure 9 (Supplementary Figure 22). Differential expression result from Zhengmix4eq data using two different analysis methods. The spearman correlation of the two methods’ statistics were greater than 0.97.*

\### Minor comments and suggestions

* p. 2 line 41 "adversary" should be changed to "adverse".

*Thank you!*

* You may want to cite a recent benchmarking paper from Germain et al 2020 (https://www.biorxiv.org/content/10.1101/2020.02.02.930578v2) in the introduction.

*Thank you. We cited this paper in the second paragraph where we divide the analysis pipeline into several chunks of processes.*

* In the last paragraph of the intro, it might be worth citing Sarkar & Stephens 2020 (https://www.biorxiv.org/content/10.1101/2020.04.07.030007v2.abstract). They further support your claim about the Poisson being good enough as a noise model.

*Thank you. We cited the paper in the first paragraph of the introduction as an example that barcoding leads to a different data structure.*

* p. 3 line 27: "standard normal distribution" should be changed to "asymptotic standard normal distribution under the null hypothesis".

*Thank you!*

* p. 3 line 57: "sequencing depth" should probably be changed to "total UMI count".

*Thank you!*

* p. 4, line 27: "protein-coding genes" should probably be changed to "non-immune genes".

*We used the gene type labels directly from the GTEx data base, and we would not conclude that protein-coding genes are non-immune genes.*

* p. 5, line 18: you may want to use the Holm correction for multiple testing since it is uniformly more powerful than the Bonferroni adjustment (https://www.jstor.org/stable/4615733) see also the documentation for p.adjust in R, where Holm is the default.

*Thank you for the suggestion. We understand that the Holm correction has higher power when our goal is to detect as many signals as possible at a given level of error measure (FWER, etc.). The context of using Bonferroni correction in our manuscript is to show that the number of genes with zero inflation decreases after clustering. The same results hold regardless of choice of multiple testing correction method, so we kept the Bonferroni example in the main text.*

* p. 5, line 34: "sufficient for cellular heterogeneity analysis" is a broad claim, I suggest this be modified to emphasize the method is intended for low counts. Now that smart-seq3 is out, I suspect there will be more UMI datasets with large counts, where zero-inflation tests could be less powerful.

*We agree, and we changed it to “We show empirically this statistic is sufficient for cellular heterogeneity analysis in many data sets with low UMI counts.”. Thank you for the suggestion.*

* p. 6, line 50: "accounts" should be "counts"

*Thank you for the correction.*

* p. 7, line 50: thank you for not hiding this scenario where the method breaks down, I appreciate your honesty!

*Thank you!*

* Table 2- should probably mention the chemistry version for the 10x datasets (eg, chromium v1/v2/v3 or gemcode). May also want to add a column saying the tissue type of each dataset, it can be guessed from second column for some, but not clear for all.

*Thank you for the comment. We added the version number as suggested. We further added the tissue information in the table. Instead, we removed the year, gene count, and cell count information from the table due to space limitation.*

* Both the Hafemeister and our (Townes) papers have been published in Genome Biology, so you may want to update the citation (currently it cites biorxiv).

*We changed the citation. Thank you for letting us know.*

* In your github readme file, you describe installing the Zhengmix4eq dataset from a URL. It may be easier to just install it from the DuoClustering2018 bioconductor package (https://www.bioconductor.org/packages/release/data/experiment/html/DuoClustering2018.html ). This would also give you quick access to their filtered versions of the data based on highly variable genes, average expression, M3Drop, etc.

*Thank you for the suggestion. We will reflect this on the next push to Bioconductor.*

**Second round of review**

**Reviewer 2**

Thanks for addressing my questions and comments.
